# Hyperoxia but not AOX expression mitigates pathological cardiac remodeling in a mouse model of inflammatory cardiomyopathy

**DOI:** 10.1038/s41598-019-49231-9

**Published:** 2019-09-04

**Authors:** Praveen K. Dhandapani, Isabel M. Begines-Moreno, Gloria Brea-Calvo, Ulrich Gärtner, Thomas G. Graeber, Gerardo Javier Sanchez, Rory E. Morty, Kai Schönig, Johanna ten Hoeve, Astrid Wietelmann, Thomas Braun, Howard T. Jacobs, Marten Szibor

**Affiliations:** 1 Faculty of Medicine and Health Technology, Tampere University, Tampere, Finland; 2 Institute of Biotechnology, University of Helsinki, Helsinki, Finland; 3 Laboratorio de Fisiopatologia Celular y Bioenergética, Universidad Pablo de Olavide, 41013 Sevilla, Spain; 4 Centro Andaluz de Biologia de Desarrollo and CIBERER, ISCIII, Universidad Pablo de Olavide-CSIC-JA, 41013 Sevilla, Spain; 5 Institute of Anatomy and Cell Biology, Justus-Liebig-University, Giessen, Germany; 6 Department of Molecular and Medical Pharmacology, Crump Institute for Molecular Imaging and UCLA Metabolomics Center, 90095 Los Angeles, USA; 7 Max-Planck Institute for Heart and Lung Research, 61231 Bad Nauheim, Germany; 8 Central Institute for Mental Health, University of Heidelberg, 68159 Mannheim, Germany

**Keywords:** Cardiomyopathies, Metabolomics, Molecular biology

## Abstract

Constitutive expression of the chemokine Mcp1 in mouse cardiomyocytes creates a model of inflammatory cardiomyopathy, with death from heart failure at age 7–8 months. A critical pathogenic role has previously been proposed for induced oxidative stress, involving NADPH oxidase activation. To test this idea, we exposed the mice to elevated oxygen levels. Against expectation, this prevented, rather than accelerated, the ultrastructural and functional signs of heart failure. This result suggests that the immune signaling initiated by Mcp1 leads instead to the inhibition of cellular oxygen usage, for which mitochondrial respiration is an obvious target. To address this hypothesis, we combined the Mcp1 model with xenotopic expression of the alternative oxidase (AOX), which provides a sink for electrons blocked from passage to oxygen via respiratory complexes III and IV. Ubiquitous AOX expression provided only a minor delay to cardiac functional deterioration and did not prevent the induction of markers of cardiac and metabolic remodeling considered a hallmark of the model. Moreover, cardiomyocyte-specific AOX expression resulted in exacerbation of Mcp1-induced heart failure, and failed to rescue a second cardiomyopathy model directly involving loss of cIV. Our findings imply that mitochondrial involvement in the pathology of inflammatory cardiomyopathy is multifaceted and complex.

## Introduction

Monocyte chemoattractant protein 1 (Mcp1), a small cytokine of the chemokine family, is expressed and secreted by pathogen-infected or damaged cells, to activate appropriate immune responses (see relevant reviews^1,2^). Its immediate effect is to promote the infiltration of immune cells, principally monocytes, and facilitate their activation as phagocytes able to attack pathogens and remove debris. Although the exact sequence of events is not fully understood, secondary signaling from immune cells promotes repair, stress-resistance and tissue remodeling, as appropriate. In the case of a cardiac infarct resulting from localized ischemia, the responses induced in this manner by Mcp1 have been shown to facilitate the survival and repair of the surrounding tissue, as well as revascularization and scar formation^3^.

Accordingly, constitutive over-expression of *Mcp1* in cardiomyocytes in the young (8–12 week-old) mouse was shown to protect against some of the consequences of myocardial infarct, notably by limiting the extent of scar formation and adverse tissue remodeling, in specific pathological paradigms^4,5^. However, in other experiments Mcp1 was found to be detrimental to recovery after a cardiac ischemic episode, based on the phenotypic improvement brought about by expressing a truncated, dominant-negative version of human MCP1^6^. Mcp1 and other cytokines have been implicated in human heart failure^7^, where their chronic activation leads to an inflammatory crisis, with generalized remodeling that eventually compromises contractile function.

The mouse line constitutively overexpressing *Mcp1* in adult cardiomyocytes (referred to hereafter as ‘the Mcp1 mouse’) shows progressive phenotypic deterioration, involving myocardial infiltration by monocytes that fail to differentiate properly, leading to an inflammatory crisis that is ultimately fatal^8^. The Mcp1 mouse is therefore an animal model for several different aspects of cardiac pathology, including ischemic preconditioning^5^, inflammatory cardiomyopathy and chronic heart failure due to adverse remodeling of the myocardium. The initial phase of tissue remodeling can be considered to be beneficial, following tissue damage due to ischemia or infection. This process is envisaged to switch metabolism at the borders of a damaged area of the myocardium to a lower energy, more glycolytic state, preserving tissue integrity during repair. On the other hand, chronic inflammatory signaling initiated by Mcp1 results in adverse remodeling of the entire myocardium. Studies in a cardiac myoblast cell-line have also suggested a direct effect of Mcp1 on cardiomyocytes, mediated by Mcp1-inducible protein (MCPIP), which elicits the activation of NADPH oxidase at the cell membrane, promoting autophagy and eventual cell death due to the resulting excessive ROS production^9^.

In order to test whether such an oxidative crisis can explain the pathology of the Mcp1 mouse, we exposed the mice to a hyperoxic regime, which would be predicted to synergize with NADPH oxidase activation. Against expectation, this treatment did not accelerate the inflammatory crisis, but instead conferred protection against it. The result suggested that the inflammatory crisis involved inhibition of the major oxygen-using machinery of the cell, namely mitochondrial oxidative phosphorylation (OXPHOS).

To test the involvement of OXPHOS, we made use of a mouse model expressing the alternative oxidase (AOX) from *Ciona intestinalis*^10^. AOX is absent from mammals but is found in most other taxa. It serves as a by-pass of the terminal steps of respiratory electron transfer from ubiquinol (CoQ) to oxygen, catalyzed by OXPHOS complex III (cIII, ubiquinol:cytochrome *c* oxidoreductase) and cIV (cytochrome *c* oxidase). In contrast to cIII and cIV, AOX is a non-proton-motive enzyme, for which reason its engagement also leads to a substantial drop in ATP yield. Importantly, AOX only becomes active under conditions where the reduction state of its primary substrate, ubiquinol, surpasses a certain threshold, typically with around 30–40% of it accumulating in the reduced form^11–13^. Thus, AOX is only engaged when the standard respiratory chain is inhibited or overloaded downstream of ubiquinol reduction, and thus its expression does not generate a global ‘short-circuit’ in mitochondrial energy metabolism.

In accordance with this, mice expressing AOX under the strong, ubiquitously active CAG promoter are viable, and exhibit essentially no physiological differences from wild-type mice when reared under standard, non-stressful conditions^10,14^. Nevertheless, in the face of specific pathological stresses, such as exposure to cyanide, a poison targeted on cIV^10,14^, AOX-expressing mice show spectacular resistance. They also resist the cardiotoxicity of a point mutation in an assembly factor for cIII^15^, and the lethal inflammatory crisis induced by lipopolysaccharide^16^. These two findings have obvious potential relevance to the Mcp1 mouse: the first because a cardiomyopathy was rescued, the second because AOX was able to attenuate macrophage activation due to respiratory chain inhibition and succinate overload. A similar process should be driven by chronic Mcp1 expression in the heart, which AOX should be able to alleviate by the same mechanism.

To address the issue of cell-type specificity we made use of two murine AOX models: the one already described^10^, which expresses AOX ubiquitously, and a second model that we constructed, enabling us to express AOX specifically in cardiomyocytes. This allowed us to assess separately whether AOX is able to overcome the lethal phenotype induced by directly crippling cIV in cardiomyocytes. Our findings, presented below, indicate that any role of mitochondrial dysfunction in the inflammatory crisis provoked by chronic Mcp1 overexpression is primarily in cells other than cardiomyocytes.

## Results

### The inflammatory crisis induced by Mcp1 can be mitigated by hyperoxia

**Figure 1:**
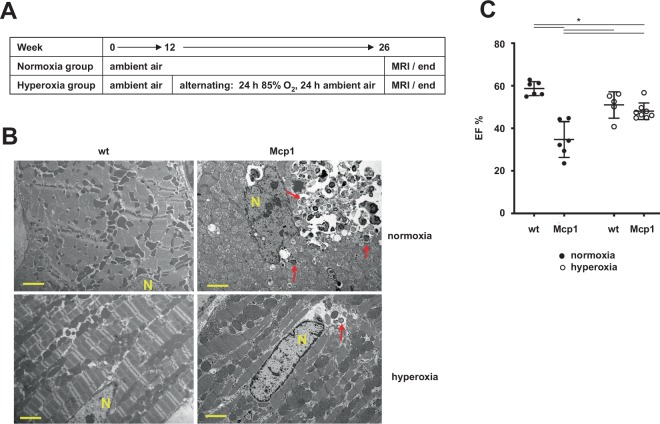
Heart failure due to Mcp1 overexpression in heart is suppressed by hyperoxia. (**A**) Summary of the experimental set-up. (**B**) Transmission electron micrographs of heart tissue from 26-week old animals of the indicated genotypes and treatments. Yellow scale-bars – 2 μm, N – nucleus, red arrows indicate structures resembling autophagosomes. The gross tissue disruption produced by Mcp1 overexpression in the heart is strongly mitigated by hyperoxia. (**C**) Cardiac ejection fraction (EF, %) of mice of the indicated genotypes (wt – wild-type, Mcp1 – overexpressing *Mcp1* in cardiomyocytes), data shown as mean ± SD, n ≥ 5 for all groups. Horizontal lines denote significantly different groups (two-way ANOVA, *post hoc* Tukey HSD test, *p* < 0.05; see Table S1). Computed from the data shown in Fig. S1C,D, using MRI conducted at 26 weeks.

In the Mcp1-overexpressing heart, tissue remodeling was marked by a progressive disruption of the tissue, involving degradation of the intracellular actomyosin network (Fig. S1A), disorganization of mitochondria, extensive intracellular vacuolation, especially in the perinuclear region (Fig. 1B), and induction of the autophagic marker LC3 (Fig. S1A). To test predictions of the previously elaborated hypothesis, that this involves an oxidative crisis in cardiomyocytes, produced by the activation of NADPH oxidase, we subjected Mcp1 mice to a hyperoxic regime (Fig. 1A). If the hypothesis is correct, hyperoxia should potentiate oxygen-dependent reactions and accelerate the loss of cardiac function. However, we instead observed a spectacular improvement, both at the ultrastructural (Fig. 1B) and functional (Fig. 1C) levels, consistent with the idea that oxygen levels over-ride rather than synergize with the effects of Mcp1 overexpression.

### Conditionally activatable AOX can be specifically expressed in mouse heart

The effect of hyperoxia suggests a completely different explanation for the phenotype produced by Mcp1 overexpression, namely that Mcp1 provokes the inhibition of the major oxygen-dependent pathway of cells, mitochondrial respiration. Respiratory inhibition at or beyond the level of cIII has already been implicated in macrophage activation^16^. Furthermore, since OXPHOS is the major producer of cellular ATP, its inhibition in cardiomyocytes could also be postulated to be part of a machinery that switches metabolism to a tissue-protective, more glycolytic state. This would result in adverse remodeling of the entire tissue, if left unchecked.

**Figure 2:**
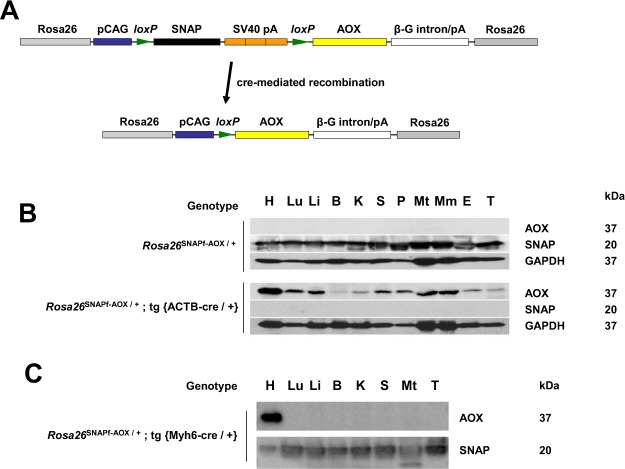
SNAPf-AOX mice express AOX when activated by tissue-specific recombination. (**A**) Schematic map of the SNAPf-AOX insert at the *Rosa26* locus (flanking regions, grey). The SNAP-tag coding sequence (black), followed by a threefold reiteration of the SV40 poly(**A**) signal (SV40 pA, orange) is flanked by loxP sites (green, directionality as shown). Cre-mediated recombination removes the entire SNAP-tag cassette, juxtaposing the synthetic CAG promoter (blue) with the AOX coding sequence (yellow), followed by the β-globin intron and poly(**A**) signal (β-G intron/pA, white). (**B**,**C**) Western blots of protein extracts from tissues of progeny mice of the indicated genotypes, from crosses between SNAPf-AOX and hemizygous mice expressing cre recombinase under the control of (**B**) the ubiquitously active β-actin (ACTB-cre) or (**C**) cardiomyocyte-specific myosin heavy-chain 6 (Myh6-cre) promoters. Tissues denoted as follows: H – heart, Lu – lung, Li – liver, B – brain, K – kidney, S – spleen, P – pancreas, Mt – thigh muscle, Mm – masseter muscle, E – eye, T – testis. Molecular weight of the detected polypeptides (kDa) inferred from the electrophoretic mobility of markers.

To test this idea, we switched to genetic models, which offer the possibility of probing mitochondrial involvement in Mcp1-induced pathology in different cell-types (whereas hyperoxia obviously affects all of them). To complement the use of the AOX mouse described above^10^ (‘global AOX’), we constructed a second mouse line (‘SNAPf-AOX’) containing a version of the AOX transgene placed downstream of a floxed copy of the coding sequence of the SNAP-tag peptide^17^. To prevent readthrough expression of AOX, the SNAP-tag cassette included three, reiterated copies of the poly(A) addition sequence from SV40 (Fig. 2A). The entire construct was inserted at the *Rosa26* locus under the control of the chimeric CAG promoter, directing ubiquitous expression, as for global AOX. However, expression is initially only of the SNAP tag. Activation of AOX expression requires Cre-mediated recombination. The insertion was verified in the donor ES cell-line by Southern hybridization and sequencing, then tracked subsequently by PCR (see Methods). Expression of the SNAP-tag peptide was verified in hemizygous SNAPf-AOX mice in all tissues tested by Western blot (Fig. 2B), whilst AOX protein was undetectable. When hemizygous SNAPf-AOX mice were crossed with a line hemizygous for ACTB-Cre, directing Cre recombinase expression ubiquitously under the control of the β-actin promoter, double transgenic progeny now expressed AOX in all tissues tested, whilst SNAP-tag expression was undetectable (Fig. 2B). Crossing hemizygous or homozygous SNAPf-AOX mice with mice expressing Cre recombinase specifically in adult cardiomyocytes under the control of the Myh6 promoter (Myh6-cre) yielded double-transgenic progeny expressing AOX uniquely in heart (Fig. 2C). Note that the SNAP tag continued to be expressed in the heart in these mice at a lower level, reflecting the fact that heart tissue contains other cell-types than just cardiomyocytes, as well as possible mosaicism due to incomplete Cre-mediated recombination-

### AOX does not rescue lethal heart failure due to Mcp1 overexpression in cardiomyocytes

**Figure 3:**
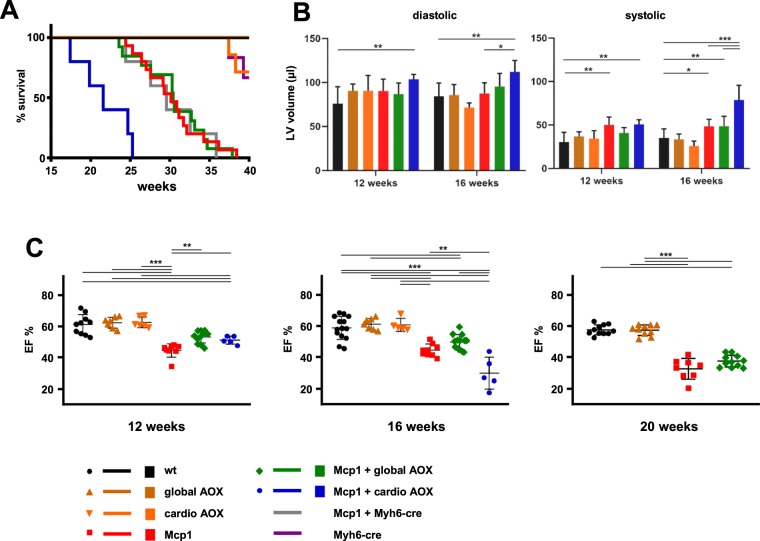
AOX expression modifies the cardiac phenotype of Mcp1 overexpressing mice. (**A**) Survival curves of mice of the indicated genotypes (wt – wild-type, Mcp1 – overexpressing Mcp1 in cardiomyocytes under the control of the Myh6 promoter; global AOX – expressing AOX constitutively; cardio AOX – expressing AOX specifically in cardiomyocytes, after activation of SNAPf-AOX by Myh6-cre, Myh6-cre – expressing only cre in adult cardiomyocytes, and combinations as shown, n ≥ 10 for all groups except those including Myh6-cre, where n ≥ 5). The survival curve for Mcp1 plus cardiomyocyte-specific AOX was significantly different from that of Mcp1 alone, or together with global AOX (*p* < 0.0001, Log-rank (Mantel-Cox) or Gehan-Breslow-Wilcoxon test, see Table S2). (**B**) Systolic and diastolic volume of the cardiac left ventricle (LV) of mice of the indicated genotypes, measured by ultrasound at 12 and 16 weeks, as indicated (means ± SD, n ≥ 5 for all groups). (**C**) Cardiac ejection fraction (EF, %) computed for individual mice of the indicated genotypes and ages, means ± SD. Horizontal lines marked with asterisks indicate significant differences (one-way ANOVA within an age point, applying Tukey’s *post hoc* HSD test; *, **, ***p < 0.05, 0.01, 0.001, respectively).

We initially tracked the survival of mice overexpressing Mcp1 in cardiomyocytes, with or without the additional presence of global or cardiomyocyte-specific AOX, alongside appropriate controls. Male Mcp1 mice died between 25 and 35 weeks of age (Fig. 3A, red trace). Global AOX expression did not alter this, nor did the Myh6-cre inducer used to activate AOX specifically in cardiomyocytes (Fig. 3A, green and grey traces, respectively), although Myh6-cre, both alone (purple trace) or together with SNAPf-AOX (orange trace, ‘cardio AOX’), did affect survival starting much later, around 40 weeks, as previously reported^18^. However, cardio AOX in combination with Mcp1 produced a clear acceleration of the lethal phenotype, with double-expressor mice dying between 16–25 weeks of age (blue trace). Cardiac parameters followed a similar pattern. Whilst mean diastolic and systolic volumes in Mcp1-overexpressing mice showed only minor deviations from the controls (Fig. 3B), computed ejection fractions (EF, Fig. 3C) revealed a progressive deterioration in heart contractile function in all Mcp1-overexpressing groups. This was already evident at 12 weeks, although both global (green diamonds) and cardio AOX (blue circles) did produce a small functional improvement at this time point, compared with Mcp1 overexpressors alone (red squares). By 16 weeks this was no longer statistically significant for global AOX. For Mcp1 mice combined with cardio AOX the EF at 16 weeks was clearly decreased compared with the other groups (Fig. 3C, blue circles), and by 20 weeks the paucity of survivors prevented a meaningful analysis. At 16 weeks, there was no evidence of cardiotoxicity in the mice expressing only cardiomyocyte-specific AOX but not Mcp1 (orange triangles). There were also no significant differences in left ventricular mass (Fig. S2A), total body weight (Fig. S2B) or in treadmill performance (Fig. S2C) between wild-type and Mcp1 mice, regardless of AOX expression, at 12 or 16 weeks.

### AOX does not alter the switch to a fetal-like gene expression program in Mcp1 mice

**Figure 4:**
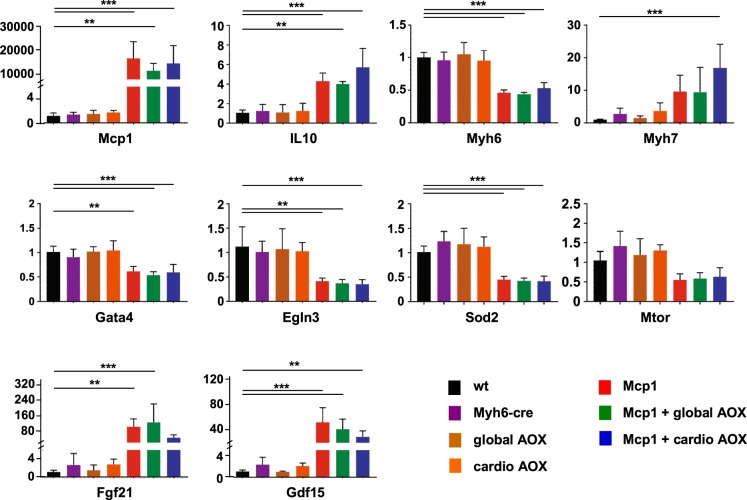
Modification of gene expression due to Mcp1/AOX expression. qRTPCR analysis of levels of the indicated RNAs in cardiac left ventricle of 12-week old mice of the genotypes shown, in each case normalized to the value for wild-type (means ± SD, n = 5 for each genotype). Horizontal lines marked with asterisks indicate significant differences between wild-type and other groups (one-way ANOVA with Tukey *post hoc* HSD test; **, ***p < 0.01, 0.001, respectively). For clarity, significant differences between other groups is not shown, but is summarized in full in Table S3.

To evaluate the combined effects of Mcp1 over-expression and AOX on heart physiology, we next analyzed the expression at the RNA level of a set of key genes involved in cardiomyocyte function, metabolic adaptation and stress signaling, serving as markers of the remodeling process. We focused on the 12-week time point, at which we had picked up the transient protective effect by AOX. We first verified the overexpression of Mcp1 (Fig. 4), which was upregulated by >4 orders of magnitude. All other changes seen were common to the three Mcp1-overexpressing groups, distinguishing them from wild-type and other controls (Fig. 4), but revealing no significant effects attributable to AOX. The mRNA for the immunomodulatory cytokine IL10 was induced several-fold, whilst cardiomyocyte markers indicated the expected shift towards a fetal-like gene expression pattern, with upregulation of Myh7, downregulation Myh6 and of Gata4, the transcription factor involved in the switch between them. This was accompanied by changes reflecting a more anaerobic metabolism, with downregulation of the hypoxia-inducible cytokine Egln3, of Sod2, responsible for detoxifying mitochondrial superoxide, and of Mtor. Two cytokines, Fgf21 and Gdf15, that have been linked to the signaling of mitochondrial dysfunction, as well as to other metabolic stresses and inflammation, were strongly induced, but this too was unaffected by AOX.

### AOX has only minimal effects on mitochondrial downregulation in Mcp1 mice

**Figure 5:**
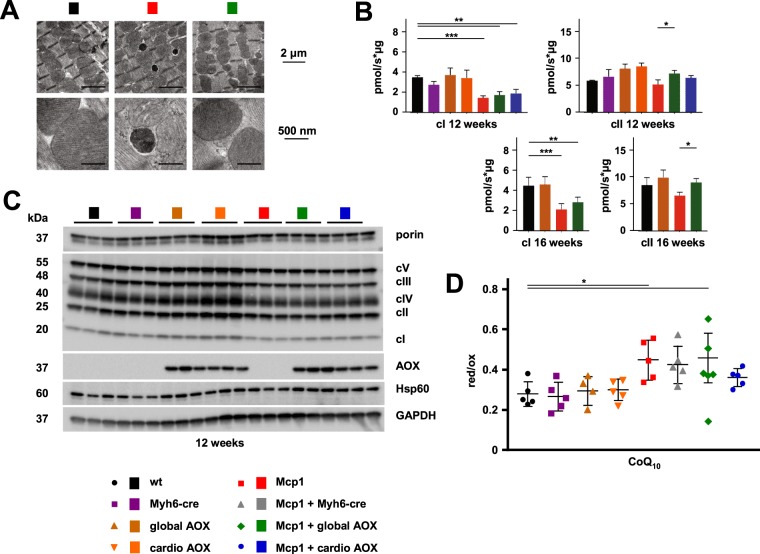
Compromised mitochondrial functions in Mcp1 mice are minimally modified by AOX. Transmission electron micrographs of cardiac left ventricle from 12-week old animals of the indicated genotypes and treatments. Scale bars as indicated: bottom images represent higher magnification of portions of those shown in the top line. The dark inclusions in close proximity to mitochondria, seen in cardiomyocytes from the Mcp1-overexpressing mice, are interpreted as secondary lysosomes. >12 such sections were analysed from mice of each genotype (2–3 sections from each of 2 blocks from 3 individuals). Multiple such inclusions were seen in the sections from Mcp1 mice, but not from controls or from mice expressing global AOX. Note also the less densely packed cristae in mitochondria from the Mcp1-overexressing mice, which was evident in all sections analysed. (**B**) Respirometry of mitochondrial suspensions from heart tissue of mice of the indicated genotypes, driven by cI- or cII-linked substrates as shown. (**C**) Representative Western blots of heart protein extracts from 12-week old mice of the indicated genotypes, probed as shown. Note that each lane represents a sample from an individual mouse, analyzed on separate gels for different proteins. Approximately equal loading was confirmed by the use of a prestained gel system (see images from these gels in Fig. S3D). For each genotype, samples from five individual mice were analyzed, giving means ± SD of densitometric signal as shown in Fig. S3A. (**D**) HPLC analysis of ratio of reduced to oxidized CoQ_10_ in hearts from 12-week old mice of the indicated genotypes. Horizontal lines denoted by asterisk (*) indicate significant differences between groups (one-way ANOVA, with Tukey’s *post hoc* HSD test, *p* < 0.05, Table S4).

The induction of Fgf21 and Gdf15 led us to investigate mitochondrial structure and function in the Mcp1 mice in more detail. Based on electron microscopy, Mcp1 overexpression (Fig. 5A, red marker) resulted in little or no disorganization of the intracellular actomyosin network at the 12-week time point. Mitochondria appeared almost normal, although the density of cristae appeared to be decreased, and we observed the presence of nearby structures that we interpret as secondary lysosomes, which were not seen in the hearts of Mcp1 mice also expressing global AOX (Fig. 5A, green marker). Respirometry revealed a clear decrease in the respiratory capacity of heart mitochondria from Mcp1 mice (Fig. 5B). Oxygen consumption on cI-linked substrates was significantly decreased at both 12 and 16 weeks (Fig. 5B). cII-linked respiration was only mildly affected at both time points, but global AOX expression significantly increased it, with cI-linked respiration showing a similar trend. The decreased respiratory capacity of Mcp1-overexpressing heart reflected a generalized, if small decrease in the abundance of respiratory chain subunits (Fig. 5C – see quantitation by densitometry in Fig. S3A), although this was less evident at 16 weeks (Fig. S3B). At both time points, AOX expression had no clear impact (Figs 5C and S3A,B). These various changes can again be interpreted as markers of the remodeling process, upon which AOX has only a minor and transient impact.

### Cardiomyocyte-specific AOX produces metabolic changes in Mcp1 mice

**Figure 6:**
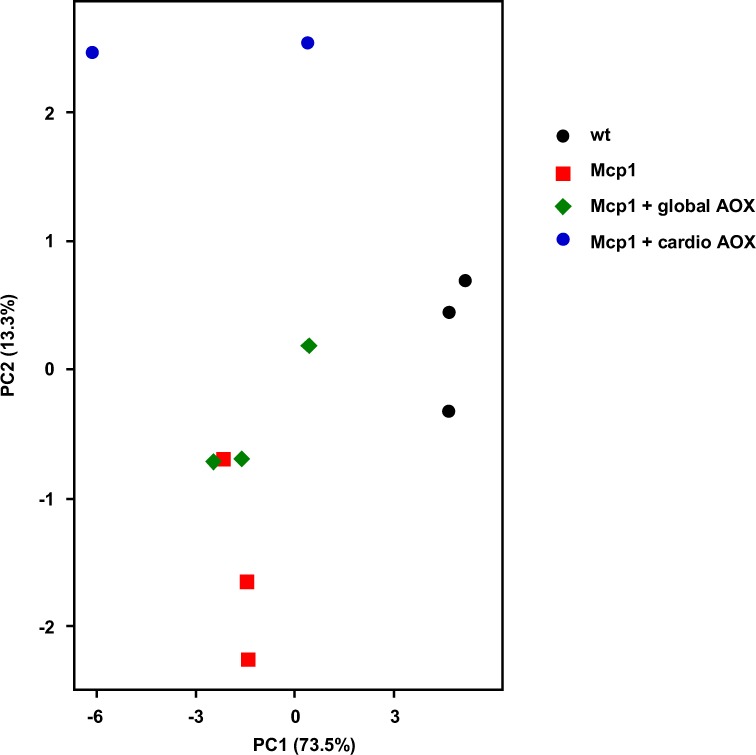
Cardiomyocyte-specific AOX expression alters cardiac metabolism of Mcp1 mice. Principal component analysis of metabolome data (^13^C-glucose tracer) from cardiac left ventricles of 12-week old mice of the indicated genotypes. The plot is based on 17 glucose metabolites that passed statistical filtering, corrected for low fractional contribution from the labelled glucose.

AOX contributes appreciably to electron flow only when the ratio of reduced to oxidized quinone rises above ~0.4. To check whether the decreased respiratory capacity of mitochondria from Mcp1- overexpressing heart had resulted in such a build-up, we measured the amounts of reduced and oxidized CoQ_9_ and CoQ_10_ in heart tissue by fast lipid extraction and HPLC. (Note that mice use both CoQ_9_ and CoQ_10_ in OXPHOS, although the former predominates). In the case of CoQ_10_ (Fig. 5D), there was a significant shift towards the reduced form in Mcp1-overexpressing heart, which was not altered by global AOX. In contrast, cardiomyocyte-specific AOX resulted in a decreased ratio of CoQ_10_(red)/CoQ_10_(ox) that was almost restored to that of wild-type (Fig. 5D). CoQ_9_ followed a similar trend (Fig. S3C), although the differences did not reach significance, and their extent was quantitatively less than for CoQ_10_. In all Mcp1 groups there was a small but significant increase in the glycolytic enzyme GAPDH (Fig. S3A), but induction of the autophagic marker LC3b-II was minimal (Fig. S3E,F) and ‘oxyblot’ revealed no evidence for widespread oxidative damage to proteins (Fig. S3G). None of these parameters was systematically affected by AOX. However, analysis of the global metabolome using a labeled glucose tracer did reveal an effect of cardiomyocyte-specific AOX already at 12 weeks. Principal component analysis (PCA) of the labeled heart metabolites (Fig. 6) showed a clear difference between control and Mcp1 mice, with the global AOX + Mcp1 mice overlapping with Mcp1 alone, but slightly shifted on the plot in the direction of wild-type, consistent with its minor, transient effect on mitochondria (Fig. 5). Mcp1 mice expressing cardio AOX were more distinct from all others (Fig. 6, blue circles), consistent with an early metabolic crisis, prefiguring the contractile dysfunction that was observed at 16 weeks (Fig. 3C). For a fuller explanation of the PCA see Fig. S4 and the associated Tables S5 and S6.

### AOX is unable to rescue heart failure caused directly by cIV deficiency

**Figure 7:**
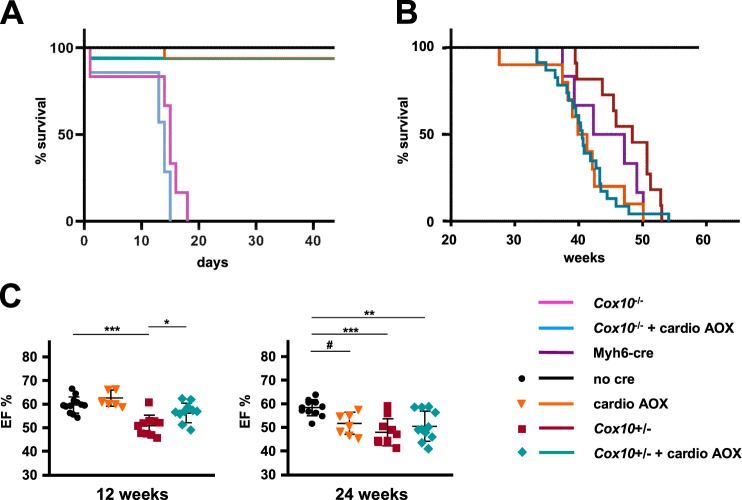
Minimal phenotypic modification by AOX, of *Cox10* heart knockout. (**A**,**B**) Survival curves of mice of the indicated genotypes: *Cox10*^−/−^ – cardiomyocyte-specific knockout of *Cox10*, i.e. mice were *Cox10*^fl/fl^ plus Myh6-cre^+/−^; *Cox10*^−/−^ + cardio AOX – cardiomyocyte-specific knockout of Cox10 plus activation of SNAPf-AOX by Myh6-cre (both heterozygous); Myh6-cre – Myh6-cre^+/−^ only, no cre – control mice lacking Myh6-cre, cardio AOX – cardiomyocyte-specific activation of SNAPf-AOX by Myh6-cre, both hemizygous; *Cox10*^+/−^ – cardiomyocyte-specific heterozygous knockout of *Cox10*, i.e. mice were *Cox10*^*+/fl*^ and Myh6-cre^+/−^; *Cox10*^+/−^ + cardio AOX – cardiomyocyte-specific heterozygous knockout of *Cox10*, i.e. mice were *Cox10*^+/*fl*^ and heterozygous for both Myh6-cre and SNAPf-AOX. n ≥ 6 for all groups. (**C**) Cardiac ejection fraction (EF, %) computed for individual mice of the indicated genotypes and ages, means ± SD, by echocardiography. Horizontal lines marked with asterisks indicate significant differences (one-way ANOVA within an age point, applying Tukey’s *post hoc* HSD test; *, **, ***p < 0.05, 0.01, 0.001, respectively).

The failure of AOX to provide a durable, functional rescue of the Mcp1-induced phenotype, and even to exacerbate it when expressed specifically in cardiomyocytes, can have various possible explanations. Despite the global downregulation of respiratory chain proteins and capacity, the data of Fig. 5B,C suggest that a key target could be cI, for which AOX does not substitute. Another possibility is that mitochondrial downregulation, although part of the tissue remodeling process, is only incidental to the pathology. The aggravation of the phenotype due to cardiomyocyte-specific AOX may also reflect the underlying dependence of contractile function on ATP. AOX, once activated, should restore redox homeostasis, as evidenced by the normalization of CoQ_10_ oxidation, (Fig. 5D), but will not replenish ATP, instead depleting it further. To test the extent to which AOX can functionally replace cIII + cIV in cardiomyocytes, we combined it with a second model in which heart failure is a direct result of mitochondrial cIV deficiency, brought about by cardiomyocyte-specific knockout of Cox10, a biosynthetic enzyme for heme *a*, an essential prosthetic group of cIV. Using Myh6-cre, we first confirmed that cardiomyocyte-specific knockout of *Cox10* was lethal at an early time point (none of the animals that survived till weaning was homozygous for the floxed *Cox10* allele and positive for Myh6-cre, out of 45 live pups, Table S7, p < 0.01, chi-squared test with Yates’ correction). We then combined cardiomyocyte-specific *Cox10* knockout with cardiomyocyte-specific AOX activation, producing essentially the same result (zero animals homozygous both for the floxed allele of *Cox10* and for Myh6-cre, whether with or without AOX, out of 80 live pups, Table S7, p < 0.001, chi-squared test, Yates’ correction). We followed a small cohort day-by-day from birth, to determine the exact timing of death (Fig. 7A), which was the same for the AOX-positive and -negative *Cox10* knockout mice. Mice with heterozygous *Cox10* knockout in cardiomyocytes, both with and without AOX, were then studied further, although this is complicated by the late-onset cardiotoxic effect of Myh6-cre on its own^18^. *Cox10* heterozygosity produced an apparent slight alleviation of the eventual lethality of Myh6-cre (Fig. 7B), whilst AOX expression exacerbated it (Fig. 7B). Conversely, at 12 weeks, heterozygous knockout of *Cox10* in cardiomyocytes caused a significant detriment to heart contractile function as measured by ejection fraction (Fig. 7C), which was mildly alleviated by AOX co-expression. By 24 weeks, this alleviation was no longer apparent, but contractile function at that time point was impaired in all groups expressing Myh6-cre (Fig. 7C). In conclusion, AOX provided little or no functional benefit as a replacement for cIII + cIV.

## Discussion

In this study, we set out to test the involvement of oxygen metabolism in the inflammatory crisis unleashed by constitutive expression of Mcp1 in cardiomyocytes. Mcp1-induced heart failure was suppressed rather than exacerbated by hyperoxia, whilst AOX had little effect, and even accelerated the lethal phenotype when expressed only in cardiomyocytes. AOX was also unable to compensate the postnatal lethality of a severe cytochrome oxidase defect in cardiomyocytes. These findings have important implications for understanding the metabolic and functional consequences of chronic inflammation in the heart.

### Why did AOX fail to alleviate the phenotype of the Mcp1 mouse?

The beneficial effects of hyperoxia in the Mcp1 model (Fig. 1) refute the involvement of NADPH oxidase (or any other oxygen-dependent enzyme) in promoting adverse remodeling, and instead suggested mitochondrial inhibition as a mechanism. However, the negative effect of cardiomyocyte-specific AOX in the Mcp1 model, and its failure to compensate for the knockout of *Cox10*, imply that restoring or maintaining mitochondrial redox homeostasis in cardiomyocytes is insufficient to sustain contractile function. Rather, our findings point toward the importance of adequate ATP production, given that AOX-supported respiration abrogates proton-pumping at both cIII and cIV, leaving only cI as an energy-yielding step. In accord with the idea that a mostly glycolytic metabolism is sufficient to maintain cardiac development and function throughout the fetal period, *Cox10* cardiac knockout pups were ostensibly indistinguishable from wild-type littermates. However, once exposed to an oxygen-rich atmosphere, they failed to thrive and died within 2–3 weeks postnatally, with AOX providing no benefit (Fig. 7A). Cardiomyocyte-specific expression of AOX in the Mcp1 mouse, which is enzymatically engaged sufficiently to lower the ratio of reduced to oxidized ubiquinone (Fig. 5D), should also lower the energetic yield of OXPHOS. This alone may be sufficient to exacerbate the contractile failure resulting from Mcp1-induced remodeling. Further analysis of the heart metabolome of the Mcp1 mice with and without cardio AOX may shed light on the processes by which the tissue responds to chronic inflammatory signaling combined with failing endogenous ATP supply.

A different possible explanation for the detrimental effect of cardiomyocyte-specific AOX in the Mcp1 mouse mirrors that elaborated earlier^13^ in the case of muscle-specific knockout of *Cox15*, another enzyme involved in heme *a* biosynthesis. In the *Cox15* model, AOX expression was found to abolish ROS signaling consequent upon respiratory chain inhibition. In skeletal muscle, this signaling is required to recruit satellite cells to replace damaged myofibrils. AOX therefore produced an exacerbation of the phenotype. Cardiac repair proceeds instead via an intracellular mechanism but if this also depends upon a mitochondrial ROS signal, e.g. elicited by oxygen in a tissue with compromised respiratory function due to chronic remodeling, cardiomyocyte-specific AOX expression would again be expected to negate it.

### Mechanism of Mcp1-induced heart failure

Although Mcp1 is overexpressed only in cardiomyocytes in the Mcp1 mouse, it is a secreted cytokine that acts directly or indirectly on a variety of cell types, including (by definition) monocytes and other immune cells, as well as fibroblasts, endothelial cells and neighboring cardiomyocytes. This complexity undoubtedly contributes to the apparently contradictory or biphasic nature of the Mcp1 response reported in the literature, which promotes vascularization and limits scar formation after an ischemic episode^5^, but eventually leads to catastrophic remodeling even in the absence of an external insult (Figs 1 and 3). Although the precise cell-type specificity of these processes is poorly understood, the models used in this study, are potentially informative. The effect of hyperoxia, given the failure of AOX to rescue, raises the possibility that direct oxygen-sensing in cardiomyocytes and/or other cells may act to negate immune-derived signals that would otherwise maintain the myocardium in a low-energy state during tissue repair, following an infectious or ischemic episode. Effective oxygen delivery could be a physiological cue that such repair is complete, and that cardiomyocytes can resume normal function. Productive versus adverse tissue modeling should reflect the balance of such signals, in which endogenous indicators of the state of the cardiomyocytes themselves should logically also play a part. The minimal effect of global AOX could therefore simply be a composite of a strongly positive effect to dampen inflammatory signaling from macrophages and a strongly negative effect arising from compromised ATP production in cardiomyocytes that just cancel one another out. To shed further light on this will require the use of combinatorial tissue expression, including the use of a macrophage-specific Cre to activate AOX expression^19^.

Note that our data do not address the question of the cell-type(s) upon which Mcp1 itself acts. The canonical targets of all chemokines are immune cells, so there is no *a priori* reason to invoke a direct effect on cardiomyocytes. Our findings with AOX strongly imply that at least one other cell-type is involved in inducing the remodeling process leading to contractile failure, for which macrophages are the obvious candidates. To pin down whether Mcp1 has any direct effect upon cardiomyocytes would require further genetic modeling, such as cell-specific knockout of the Mcp1 receptor CCR2.

### Other processes may influence global and cardiomyocyte-specific AOX phenotypes

There remain other possible explanations for the phenotypic difference between global AOX and cardiomyocyte-specific AOX expression in the Mcp1 mouse, which should be considered. One is that Myh6-cre is not sufficiently expressed in all cells to produce a uniform genomic rearrangement. In SNAPf-AOX mice also expressing Myh6-cre, some residual SNAP-tag expression was seen in heart (Fig. 2C), which is attributable to expression in other cell types than cardiomyocytes. If, however, the SNAPf-AOX insert in a minor fraction of cardiomyocytes were not recombinationally resolved, the myocardium would be a mosaic of AOX-positive and -negative cells. These would manifest different types of energetic metabolism, potentially compromising their ability to contract in unison. Mosaicism of this kind has been previously reported for several Cre recombinase lines^20–24^, including some directing myocardial expression^25^. It can also be background-dependent^22^. Myh6-cre appears to be efficient^26^, but there is some reported mosaicism in the timing of its expression^27^ that may be relevant. However, even if present, metabolic heterogeneity of cardiomyocytes may not have a major functional effect^28^.

Another possibility is that constitutive AOX expression from even before fertilization enables energy metabolism to be epigenetically adapted to its presence^29^. In contrast, Myh6-cre is only expressed when the adult-type myosin heavy chain 6 would normally start to replace the fetal-type myosin heavy chain 7. Thus, AOX would be expressed in cardiomyocytes only from late fetal stages on, which may conflict with the epigenetic programming of metabolism set earlier in development.

### Perspectives

Although a key finding of the current study, it is difficult to follow up on the observation that hyperoxia counteracts heart failure in the Mcp1 mouse, without recourse to the kinds of genetic models described here. However, combining it with cell-specific activation of AOX, deletion of CCR2 and manipulation of other oxygen-sensitive enzymes and cytokines should now be a useful approach, helping to define where it acts and by what mechanism. One attractive idea is that Mcp1 induces a hypoxia-like response, for example by HIF activation, and this is counteracted by high oxygen levels, but not by AOX. In principle, this should be testable. Another possibility, consistent with this idea and with our observation that Mcp1 does provoke a clear respiratory defect (Fig. 5), is that inhibition or down-regulation of cIII + cIV is accompanied by a down-regulation also of cI. In a previous study of cells cultured in glucose-rich medium we inferred precisely such a process, resulting from drug-inhibition of cIII or cIV, leading to a shutdown of cI which AOX was unable to relieve^30^. We inferred that an unknown signal emanating directly from cIII or cIV must impose such a regulation on cI, rather than simply the interruption of respiratory electron flow to oxygen. Introducing a by-pass of cI via the alternative NADH dehydrogenase Ndi1 from yeast was able to overcome this blockade^30^. However, implementing both by-passes simultaneously in the Mcp1 model *in vivo* is very unlikely to succeed, since it would completely short-circuit mitochondrial ATP production. Although it can work in cultured cells, it failed in an OXPHOS disease model in *Drosophila*^31^, instead producing lethality. To test it in the Mcp1 mouse we would need first to elucidate its mechanistic basis in cultured cells, then target the relevant molecular machinery. The action of Mcp1 is evidently complex, and will require much deeper knowledge of macrophage as well as cardiomyocyte biology, before its roles in cardiac disease can be properly understood.

## Methods

### Laboratory animals, procedures and ethical permits

Transgenic mice overexpressing Mcp1 specifically in cardiomyocytes, directed by the *Myh6* (*Myhca*) promoter^32^, here described as ‘Mcp1 mice’, were supplied by Pappachan Kolattukudy, University of Central Florida. *Cox10*^fl/fl^ mice^33^ were supplied by Carlos Moraes, University of Miami. Mice expressing Myh6-cre (Stock No: 011038) and ACTB-cre (Stock No: 019099) were purchased from Jackson Laboratories. For the initial immunohistochemistry and hyperoxia experiments, Mcp1 mice were maintained in the original background (FVB) in which they were supplied, using non-transgenic FVB littermates as controls. For all other experiments, Mcp1, *Cox10*^fl/fl^, Myh6-cre and ACTB-cre mice were backcrossed over ≥8 generations to C57Bl6/JOlaHsd (Envigo), in which the previously described *AOX*^*Rosa26*^ strain^10^ was also maintained. SNAPf-AOX mice, containing a floxed SNAP-tag expression cassette upstream of the AOX coding region as described in this paper, were created and backcrossed to strain C57Bl6/JOlaHsd using essentially the same methods as described previously for the *AOX*^*Rosa26*^ mice^10^. All animals were maintained and treated according to the regulations of the competent animal welfare agencies, and all of the experimental protocols were approved according to the following permits: for experiments conducted in Germany, Regierungspräsidium Darmstadt (animal handling permit V54-19c20/15-B2-293) and for experiments conducted in Finland, ELLA (Animal Experimental Board in Finland, ethical permit: ESAVI/8766/04.10.07/2015). Mice were housed in a humidity and temperature-regulated animal facility with food and water provided *ad libitum*, and were euthanized at the end of the experiment by cervical dislocation, in some cases with prior exposure to CO_2_. Humane end points were followed as described in the animal permits but, in addition, signs of prolonged immobility and/or consistent body shivers were considered as indicators of severe stress and the mice were duly terminated. Except where stated, experiments used males only, in order to minimize the total number of animals required. To test the effects of hyperoxia, mice were transferred at 12 weeks of age to an 85% O_2_ environment in a closed chamber, alternating at 24 h intervals with ambient air^34^ (see Fig. 1A).

### Behavioral and physiological analyses

Cardiac function was assessed by MRI^35^ using OsiriX software (Pixmeo), or by echocardiography using a Vevo 2100 imager^10^, as indicated in the relevant figures. Body weight measurement and endurance (treadmill) experiments were performed as previously described^10^, except that, for the treadmill procedure, the total distance run was recorded instead of time, although the experimental setup was otherwise identical.

### Genotyping

All animals used in the experiments were PCR-genotyped for all relevant loci, using crude ear-punch DNA as described previously^10^, with the primer sets indicated in Table S8.

### Transmission electron microscopy

A small piece of left ventricle (from mice of both sexes) was excised and stored at 4 °C in a buffer containing 1.5% glutaraldehyde, 1.5% formaldehyde, 0.15 M HEPES/KOH, pH 7.4 until embedding and sectioning. For epoxy resin embedding, the samples were post-fixed in 1% osmium tetroxide, stained *en bloc* in half-saturated uranyl acetate, dehydrated in an ascending ethanol series and finally embedded in Agar 100 (Agar Scientific Ltd). Ultrathin sections (60–70 nm) were cut using an Ultracut E ultramicrotome (Leica-Reichert) and examined by transmission electron microscopy (Zeiss EM900 digital). Digital images were captured with a slow-scan 2 K CCD camera (TRS, Moorenweis, Germany). For each animal, images were generated from 2–3 sections from each of 2 heart-tissue blocks, using 3 individual animals of each genotype. An alternative procedure used in some experiments involved fixation in 3% glutaraldehyde buffered with 0.1 M sodium cacodylate, embedding in Epon medium (Sigma-Aldrich) and staining with uranyl acetate and lead citrate. These samples were viewed and photographed in a Philips CM 10 electron microscope.

### Immunohistochemistry

Tissue samples were snap-frozen and stored in liquid N_2_ until cryo-sectioning. Sections were stained for LC3 (Cell Signaling antibody #2775), and counterstained for DNA with DAPI (Bioquest #17507) and for F-actin with phalloidin (Sigma-Aldrich #P5282). Sections were imaged using a Leica SP2 laser-scanning confocal microscope.

### RNA isolation and qRTPCR

Total RNA was extracted from left ventricle tissue using bead homogenization in 1 ml (>10 volumes) of TRIzol reagent (Ambion). After incubation for 10 min at room temperature, samples were gently extracted with 0.2 volumes of chloroform and centrifuged at 12,000 *g*_*max*_ for 15 min at 4 °C. The upper (aqueous) phase was collected and RNA precipitated with an equal volume of isopropanol, followed by centrifugation at 12,000 *g*_*max*_ for 15 min at 4 °C. The pellet was washed with 70% ethanol, air dried, resuspended in 20 μl nuclease-free water and quantified by NanoDrop spectrophotometry (ThermoFisher Scientific). Samples were DNase treated and reverse transcribed using the Maxima First Strand cDNA Synthesis Kit for RT-qPCR (ThermoFisher Scientific), following the manufacturer’s recommended procedure. The final 20 μl reaction volume was diluted 1:10 with nuclease-free water and 5 μl aliquots were used for qRTPCR, using the primers listed in Table S8 and the SensiFAST SYBR® No-ROX kit (Bioline), with initial denaturation at 95 °C for 2 min, followed by 40 cycles of denaturation (95 °C, 5 s), annealing and synthesis (60 °C, 30 s). After checking melting curves, samples were analyzed using a plate reader and RNA levels computed, applying the ddCT algorithm where CT values were first normalized to those of housekeeping gene *Rps18* gene, then to the wild-type control, and finally used as exponents to generate 2^−ddCT^.

### Protein extraction and Western blotting

Protein isolation and Western blotting were conducted essentially as described previously^10^. 10 μg protein aliquots were electrophoresed on 4–20% TGX stain-free gels (Bio-Rad) and transferred to PVDF membranes using Trans-Blot® Turbo^TM^ PVDF Transfer pack (Bio-Rad). Primary antibodies and their dilutions were as specified previously^10^, plus the following: porin (Abcam ab15895, rabbit polyclonal, 1:1,000), OXPHOS cocktail (Abcam ab110413, formerly Mitosciences ms604, mouse monoclonals for single subunits of each OXPHOS complex, namely NDUFB8, SDHB, UQCRC2, MTCOI and ATP5A, 1:250), AOX (21^st^ Century Biochemicals, customized rabbit polyclonal, 1:40,000), HSP60 (Abcam ab46798, rabbit polyclonal, 1:20,000), GAPDH (Cell Signaling #2118, rabbit polyclonal, 1:1,000) and LC3b (Novus NB600-1384, rabbit polyclonal, 1:1,000). Secondary antibodies and their dilutions were: goat anti-mouse IgG (Jackson ImmunoResearch #115-035-146, 1:10,000) and goat anti-rabbit IgG (Jackson ImmunoResearch #111-035-144, 1:20,000). Blot images have been cropped, rotated and framed where appropriate, and optimized for contrast and brightness, with reprobings of the same gel or different regions of the same gel shown as distinct image panels separated by white spacers. No other manipulations were introduced.

### Mitochondrial isolation and respirometry

Mitochondria were isolated from heart and used for respirometry, essentially as described previously^10^. Flux values [pmol O_2_/(s × ml)] obtained from the traces were normalized to the amount of mitochondrial protein. The activity of AOX (in samples derived from AOX-expressing animals only), as well that of cytochrome *c* oxidase in all samples, was verified, even though these measurements are not shown in the figures.

### Assays of the reduction state of ubiquinone (CoQ)

The ratio of reduced to oxidized CoQ was determined by a rapid lipid extraction method. 10 to 15 mg of heart tissue (both sexes) was homogenized with a manual micro-pestle in 20 mM potassium phosphate buffer, pH 7.5. Debris was removed by maximum speed centrifugation at 4 °C. 100 μl of sample was mixed by vortexing with 330 μl of n-propanol in the presence of 0.5 mM β-mercaptoethanol. After a further centrifugation at maximum speed to remove any remaining debris the clean lipid extracts were injected into a reverse-phase Beckmann 166 HPLC system, equipped with a C18 column (5 μm, 150 × 4.6 mm) and column oven set up at 40 °C. The mobile phase, flow rate and gradient settings were as described previously^36^. CoQ detection was performed by a Coulochem III ESA electrochemical detector linked to the HPLC system, and conditioning cell was set up before the injection valve in order to maintain the oxidation state of the sample analytes. For redox status analysis, oxidized and reduced peaks of both CoQ_9_ and CoQ_10_ were quantified and their ratio calculated. Commercial CoQ_6_ was used as an internal standard in all runs.

### Metabolite analysis

Food was withdrawn 4–6 h before the experiment, whilst water supply *ad libitum* was maintained. Mice (both sexes) were anesthetized with 4% isoflurane before injecting either U-^13^C6 labeled (CLM-1396, Larodan) or ^12^C6 (492167, Sigma-Aldrich) glucose solution (2 g/kg bodyweight). 15 min after injection, mice were sacrificed by cervical dislocation and a piece of left ventricle tissue was collected and snap-frozen in liquid nitrogen. All samples were stored at −80 °C until processing.

Frozen heart tissues were homogenized in 1 ml cold (−80 °C) 80% methanol on dry ice. After clarification by centrifugation for 5 min at 4 °C, the supernatant was transferred to an Eppendorf tube. The remaining pellet was resuspended in RIPA protein lysis buffer and protein concentration was determined (by the BCA method). A fraction of the supernatant corresponding to 5 μg protein from each sample was transferred to a glass vial and 5 nmol norvaline was added as an internal control. Metabolite extracts were dried using an EZ-2 Elite evaporator (SP Scientific) and stored at −80 °C. Samples were analyzed with an UltiMate 3000RSLC HPLC (Thermo Scientific) coupled to a Q Exactive mass spectrometer (Thermo Scientific). After resuspension in 50% acetonitrile, one-tenth of each sample was loaded onto a Luna NH2 (3 μm 100 A, 150 mm × 2 mm, Phenomenex) column. Separation was achieved using (A) 5 mM ammonium acetate (pH 9.9) and (B) acetonitrile at a flowrate of 200 μl/min. A gradient from 15% to 95% of (A) over 18 min was utilized, followed by an isocratic step at 95% (A) for 9 min and re-equilibration to the initial 15% (A) for 7 min. The Q Exactive was run with polarity switching (+3.50 kV/−3.50 kV) in full-scan mode with an m/z range of 65–975. Metabolites were quantified with TraceFinder 4.1 (ThermoFisher Scientific) using accurate mass measurements (≤3 ppm) and expected retention times established with pure standards. Data analysis, including principal component analysis and hierarchical clustering was performed using in-house scripts in the statistical language R. Statistical differences were determined by one-way ANOVA testing.

### Statistical analysis

Data groups were compared by one-way ANOVA, with *post hoc* Tukey HSD (multiple comparisons) test or, where, two factors were considered, by two-way ANOVA, followed by *post hoc* Tukey HSD (multiple comparisons) test where interaction was detected or where more than two levels were considered for a given significant factor. Survival curves were compared by the log-rank (Mantel-Cox) and Gehan-Brewslow-Wilcoxon tests, all using GraphPad Prism software. The significance of progeny-class numbers obtained in *Cox10* crosses was assessed by the chi-squared test with Yates’ correction for continuity, implemented using an online tool^37^. Statistical analyses presented in Supplementary Tables give exact *p* values, which are generally quoted in figures and legends using a conventional *, **, *** nomenclature to denote appropriate thresholds.

## Supplementary information

Mcp1 supplementary

S1

S2

S3

S4

S5

S6

S7

S8

## Data Availability

The authors are willing to share all materials reported in this paper for academic or non-profit research. All data generated or analysed during this study are included in this published article (and its Supplementary Information files); source data is freely available on request.

## References

[1] Gu L (1997). *In vivo* properties of monocyte chemoattractant protein-1. J. Leukoc. Biol..

[2] Deshmane SL, Kremlev S, Amini S, Sawaya BE (2009). Monocyte chemoattractant protein-1 (MCP-1): an overview. J. Interferon Cytokine Res..

[3] Frangogiannis NG, Smith CW, Entman ML (2002). The inflammatory response in myocardial infarction. Cardiovasc. Res..

[4] Martire A (2003). Cardiac overexpression of monocyte chemoattractant protein-1 in transgenic mice mimics ischemic preconditioning through SAPK/JNK1/2 activation. Cardiovasc. Res..

[5] Morimoto H (2006). Cardiac overexpression of monocyte chemoattractant protein-1 in transgenic mice prevents cardiac dysfunction and remodeling after myocardial infarction. Circ. Res..

[6] Hayashidani S (2003). Anti-monocyte chemoattractant protein-1 gene therapy attenuates left ventricular remodeling and failure after experimental myocardial infarction. Circulation.

[7] Yndestad A (2007). Role of inflammation in the progression of heart failure. Curr. Cardiol. Rep..

[8] Kolattukudy PE, Niu J (2012). Inflammation, endoplasmic reticulum stress, autophagy, and the monocyte chemoattractant protein-1/CCR2 pathway. Circ. Res..

[9] Younce CW, Kolattukudy PE (2010). MCP-1 causes cardiomyoblast death via autophagy resulting from ER stress caused by oxidative stress generated by inducing a novel zinc-finger protein, MCPIP. Biochem. J..

[10] Szibor M (2017). Broad AOX expression in a genetically tractable mouse model does not disturb normal physiology. Dis. Models Mech..

[11] Dry IB, Moore AL, Day DA, Wiskich JT (1989). Regulation of alternative pathway activity in plant mitochondria: nonlinear relationship between electron flux and the redox poise of the quinone pool. Arch. Biochem. Biophys..

[12] Hoefnagel MHN, Wiskich JT (1998). Activation of the plant alternative oxidase by high reduction levels of the Q-Pool and pyruvate. Arch. Biochem. Biophys..

[13] Dogan SA (2018). Perturbed redox signaling exacerbates a mitochondrial myopathy. Cell Metab..

[14] El-Khoury R (2013). Alternative oxidase expression in the mouse enables bypassing cytochrome c oxidase blockade and limits mitochondrial ROS overproduction. PLoS Genet..

[15] Rajendran J (2019). Expression of alternative oxidase prevents lethal mitochondrial cardiomyopathy in a mouse model of complex III deficiency. EMBO Mol. Med..

[16] Mills Evanna L., Kelly Beth, Logan Angela, Costa Ana S.H., Varma Mukund, Bryant Clare E., Tourlomousis Panagiotis, Däbritz J. Henry M., Gottlieb Eyal, Latorre Isabel, Corr Sinéad C., McManus Gavin, Ryan Dylan, Jacobs Howard T., Szibor Marten, Xavier Ramnik J., Braun Thomas, Frezza Christian, Murphy Michael P., O’Neill Luke A. (2016). Succinate Dehydrogenase Supports Metabolic Repurposing of Mitochondria to Drive Inflammatory Macrophages. Cell.

[17] Regoes A, Hehl AB (2005). SNAP-tag mediated live cell labeling as an alternative to GFP in anaerobic organisms. BioTechniques.

[18] Pugach EK, Richmond PA, Azofeifa JG, Dowell RD, Leinwand LA (2015). Prolonged Cre expression driven by the α-myosin heavy chain promoter can be cardiotoxic. J. Mol. Cell. Cardiol..

[19] Shi J, Hua L, Harmer D, Li P, Ren G (2018). Cre driver mice targeting macrophages. Methods Mol Biol..

[20] Bergqvist I, Eriksson B, Eriksson M, Holmberg D (1998). Transgenic Cre recombinase expression in germ cells and early embryogenesis directs homogeneous and ubiquitous deletion of loxP-flanked gene segments. FEBS Lett..

[21] Le YZ (2006). Mouse opsin promoter-directed Cre recombinase expression in transgenic mice. Mol. Vis..

[22] Iacovelli J (2011). Generation of Cre transgenic mice with postnatal RPE-specific ocular expression. Invest. Ophthalmol. Vis. Sci..

[23] Bao J, Ma HY, Schuster A, Lin YM, Yan W (2013). Incomplete cre-mediated excision leads to phenotypic differences between Stra8-iCre; Mov10l1(lox/lox) and Stra8-iCre; Mov10l1(lox/Δ) mice. Genesis.

[24] Frenz S (2015). Mosaic pattern of Cre recombinase expression in cochlear outer hair cells of the Brn3.1 Cre mouse. Neuroreport.

[25] Breckenridge R, Kotecha S, Towers N, Bennett M, Mohun T (2007). Pan-myocardial expression of Cre recombinase throughout mouse development. Genesis.

[26] Yin Z (2008). Heart-specific ablation of Prkar1a causes failure of heart development and myxomagenesis. Circulation.

[27] Zhu Y (2013). Mechanistic target of rapamycin (Mtor) is essential for murine embryonic heart development and growth. PLoS One.

[28] Villa Del Campo C, Clavería C, Sierra R, Torres M (2014). Cell competition promotes phenotypically silent cardiomyocyte replacement in the mammalian heart. Cell Rep..

[29] Etchegaray JP, Mostovslavsky R (2016). Interplay between metabolism and epigenetics: a nuclear adaptation to environmental changes. Mol. Cell.

[30] Cannino G (2012). Glucose modulates respiratory complex I activity in response to acute mitochondrial dysfunction. J. Biol. Chem..

[31] Kemppainen, K. K., Kemppainen, E. & Jacobs, H. T. The alternative oxidase AOX does not rescue the phenotype of tko25t mutant flies. *G3 (Bethesda)* 4, 2013–2021 82014). 10.1534/g3.114.013946 PMC4199707 25147191

[32] Kolattukudy PE (1998). Myocarditis induced by targeted expression of the MCP-1 gene in murine cardiac muscle. Am. J. Pathol..

[33] Diaz F, Thomas CK, Garcia S, Hernandez D, Moraes CT (2005). Mice lacking COX10 in skeletal muscle recapitulate the phenotype of progressive mitochondrial myopathies associated with cytochrome c oxidase deficiency. Hum Mol Genet..

[34] Alejandre-Alcázar MA (2007). Hyperoxia modulates TGF-beta/BMP signaling in a mouse model of bronchopulmonary dysplasia. Am. J. Physiol. Lung Cell Mol. Physiol..

[35] Ziebart T (2008). Sustained persistence of transplanted proangiogenic cells contributes to neovascularization and cardiac function after ischemia. Circ. Res..

[36] Rodríguez-Aguilera JC, Cortés AB, Fernández-Ayala DJ, Navas P (2017). Biochemical assessment of Coenzyme Q_10_ deficiency. J. Clin. Med..

[37] Preacher, K. J. Calculation for the chi-square test: An interactive calculation tool for chi-square tests of goodness of fit and independence [Computer software], http://quantpsy.org (2001).

