# Supplementary material for: Hyperoxia but not AOX expression mitigates pathological cardiac remodeling in a mouse model of inflammatory cardiomyopathy
Source: Sci Rep. 2019 Sep 4;9:12741. doi: 10.1038/s41598-019-49231-9 (PMC6726756; doi:10.1038/s41598-019-49231-9)
Supplement: Supplementary file 1 — Mcp1 supplementary [file 41598_2019_49231_MOESM1_ESM.pdf]

Hyperoxia but not AOX expression mitigates pathological cardiac remodeling in a mouse model of inflammatory cardiomyopathy

Praveen K. Dhandapani, Isabel M. Begines-Moreno, Gloria Brea-Calvo,

Ulrich Gärtner, Thomas G. Graeber, Gerardo Javier Sanchez,

Rory E. Morty, Kai Schönig, Johanna ten Hoeve, Astrid Wietelmann,

Thomas Braun, Howard T. Jacobs & Marten Szibor

SUPPLEMENTARY DATA

LEGENDS TO SUPPLEMENTARY FIGURES

Figure S1

Supplementary data on the phenotype of Mcp1-overexpressing mice and effects of hyperoxia

(A) Immunohistochemistry of hearts of mice over-expressing *Mcp1* specifically in cardiomyocytes (denoted MCP1-TG) and wild-type littermate controls, stained as indicated with antisera for the autophagy-related marker LC3, for F-actin and with DAPI for DNA, at the ages indicated. Mcp1-overexpressing heart progressively accumulates LC3-positive puncta. Scale bars (30 μm) as indicated. (B) Total body weight, (C) cardiac left-ventricular diastolic and (D) systolic left-ventricular volume of mice of the indicated genotypes (WT – wild-type, MCP1 – overexpressing *Mcp1* in cardiomyocytes), means $\pm$ SD, n $\geq$ 5 for all groups. Horizontal lines denote significantly different groups (two-way ANOVA, *post hoc* Tukey HSD test, $p < 0.05$). The ejection fraction of individual mice in this experiment, as shown in Fig. 1C, was computed from these data, which were based on MRI conducted at 26 weeks, prior to termination of the experiment. Normoxia – ambient air, Hyperoxia – oxygenation routine as shown in Fig. 1A. Note that aligned microscopy images that are not from the same field are delineated by white spacers.

Figure S2

Supplementary data on physiological parameters of Mcp1/AOX-expressing mice

(A) Left ventricular (LV) mass determined by echocardiography of mice of the indicated genotypes, at 12 and 16 weeks of age. Means $\pm$ SD, n $\geq$ 6 for all groups. (B) Total body weight and (C) distance of endurance running on treadmill, of mice of the indicated genotypes, at 12 and 16 weeks of age. Means $\pm$ SD, n $\geq$ 5 for all groups.

Figure S3

Supplementary data on biochemical parameters of Mcp1/AOX-expressing mice

(A) Densitometric analysis of Western blots for proteins belonging to each of the indicated complexes (cI –Ndufb8, cII – Sdhb, cIII – Uqcrc2, cIV – Mtco1, cV – Atp5a), and for porin and Hsp60, based on Western blots shown in Fig. 5C. Each 10 μg sample was from the left ventricle of an individual 12-week old mouse, pooled according to genotype as indicated, to generate means $\pm$ SD (n $\geq$ 5 for all groups), after normalizing to the signal intensity of wild-type. Since standard loading controls (GAPDH, Hsp60, porin) were investigated as part of the experiment, approximately equal loading was verified by the use of internally stained gels, examples of which are shown in (D), for the blot strips shown in Fig. 5C. Note that multiple gels were run to enable unambiguous detection of proteins of similar size. Values are normalized to the mean densitometric signal for wild-type controls (black bars) for each polypeptide. Asterisks above horizontal bars denote significance differences between groups (one-way ANOVA, *, **, *** – $p < 0.05$, 0.01, 0.001, respectively). (B) Equivalent, representative blot for samples from 16-week old mice as indicated. (C) HPLC analysis of ratio of reduced to oxidized CoQ_9 in hearts from 12-week old mice of the indicated genotypes; equivalent to the data for CoQ_{10} for the same samples, shown in Fig. 5D, but with quantitatively lower values and without significant differences (one-way ANOVA, Tables S4), though showing the same trends as seen for CoQ_{10}. (E) Western blot for autophagy-related protein LC3b of the same protein samples from 12-week old mice as analyzed in Fig. 5C. The abundance of the lipid-conjugated form (LC3b2), considered as a marker for autophagy, appears elevated in Mcp1 overexpressing mice, although this was mostly non-significant, as shown in (F). A similar trend was seen at 16 weeks. However, much more extensive analysis would be needed to elucidate the role, if any, of autophagy in cardiac remodeling in the Mcp1 mouse. (F) Densitometric analysis of the Western blot of Fig. S3E (and parallel blots) indicating the trend towards increased LC3b expression in the Mcp1 mouse. Normalized to wild-type, as in (A). (G) 'Oxyblot' Western analysis of total protein oxidation in left ventricle of 16-week old Mcp1/AOX mice and controls, as indicated. Mcp1

overexpression in heart does not result in a substantial quantitative or qualitative change in protein oxidation at this time point. Note that aligned Western blot images that are not from the same gel or probing, or are non-adjacent in the same gel, are delineated by white spacers.

Figure S4

Extended explanation of principal component analysis (PCA) of metabolome data

Page 1: scree plot showing fractional contribution by the principal components

Page 2: scatter plot matrix for PC1-5

Page 3: the PCA used for plotting Figure 6 (left) and correlation circle plot of the metabolites included in the analysis. PC1 generally separates wild-type from all Mcp1 samples, where metabolites on the left generally have more 13C labeling from the input glucose tracer in the Mcp1 samples compared with wild-type. The implication is that Mcp1 overexpression has switched metabolism towards higher glucose-based contribution to the synthesis of these common metabolites (most of them being amino acids and TCA cycle intermediates). This might be considered as part of a switch towards a more fetal-like phenotype, with greater reliance on glycolysis for providing carbon skeletons for biosynthesis. PC2 generally distinguishes the 'Heart AOX + Mcp1' from all other samples, where the 4 metabolites at the bottom (right) generally have less 13C labeling from the input glucose in the 'Heart AOX + Mcp1' samples. These metabolites include glucose itself and earlier intermediates/side products of glycolysis, and may represent a signature of an impending metabolic crisis. See Tables S5 and S6 for source data.

A

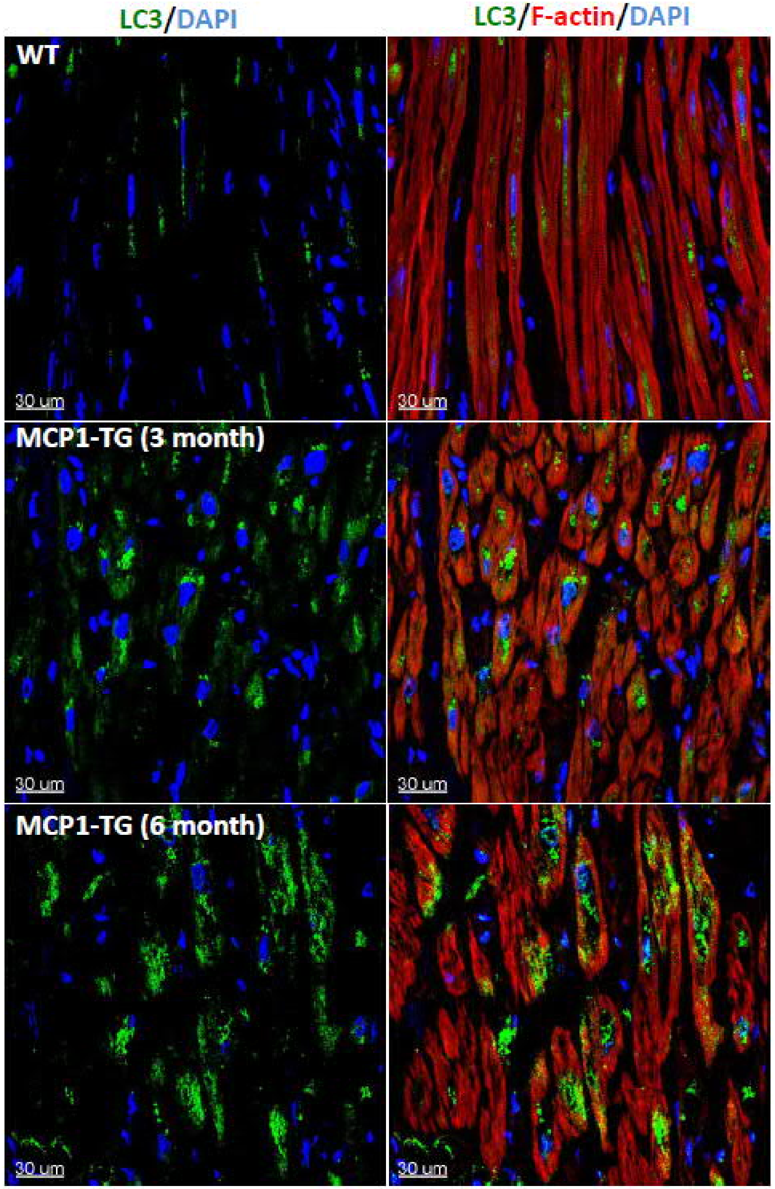

Dhandapani et al, Figure S1, page 1 of 2

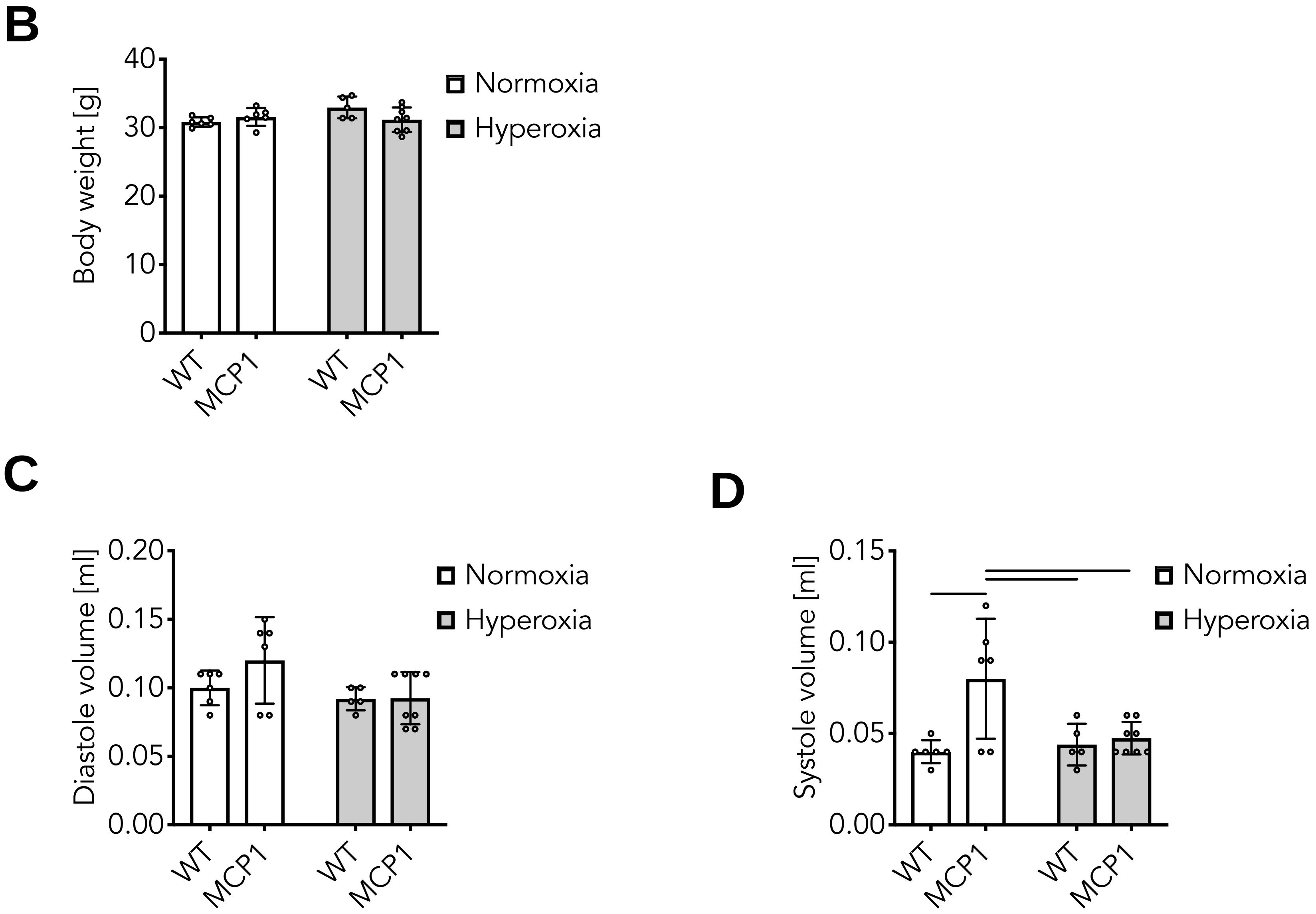

B
Body weight [g]
0
10
20
30
40
Normoxia
Hyperoxia
WT
MCP1
WT
MCP1
C
Diastole volume [ml]
0.00
0.05
0.10
0.15
0.20
Normoxia
Hyperoxia
WT
MCP1
WT
MCP1
D
Systole volume [ml]
0.00
0.05
0.10
0.15
Normoxia
Hyperoxia
WT
MCP1
WT
MCP1

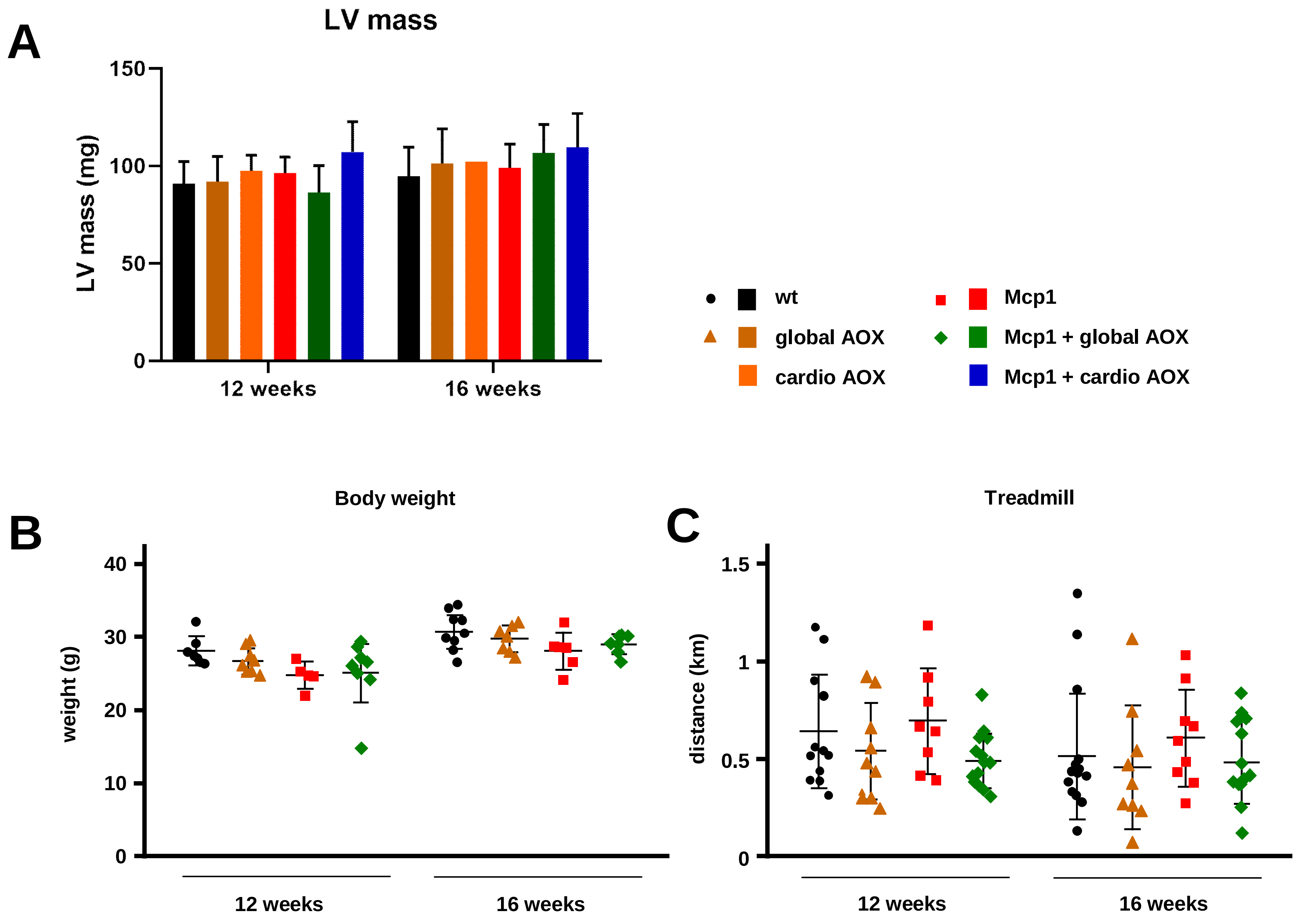

Dhandapani et al, Figure S2

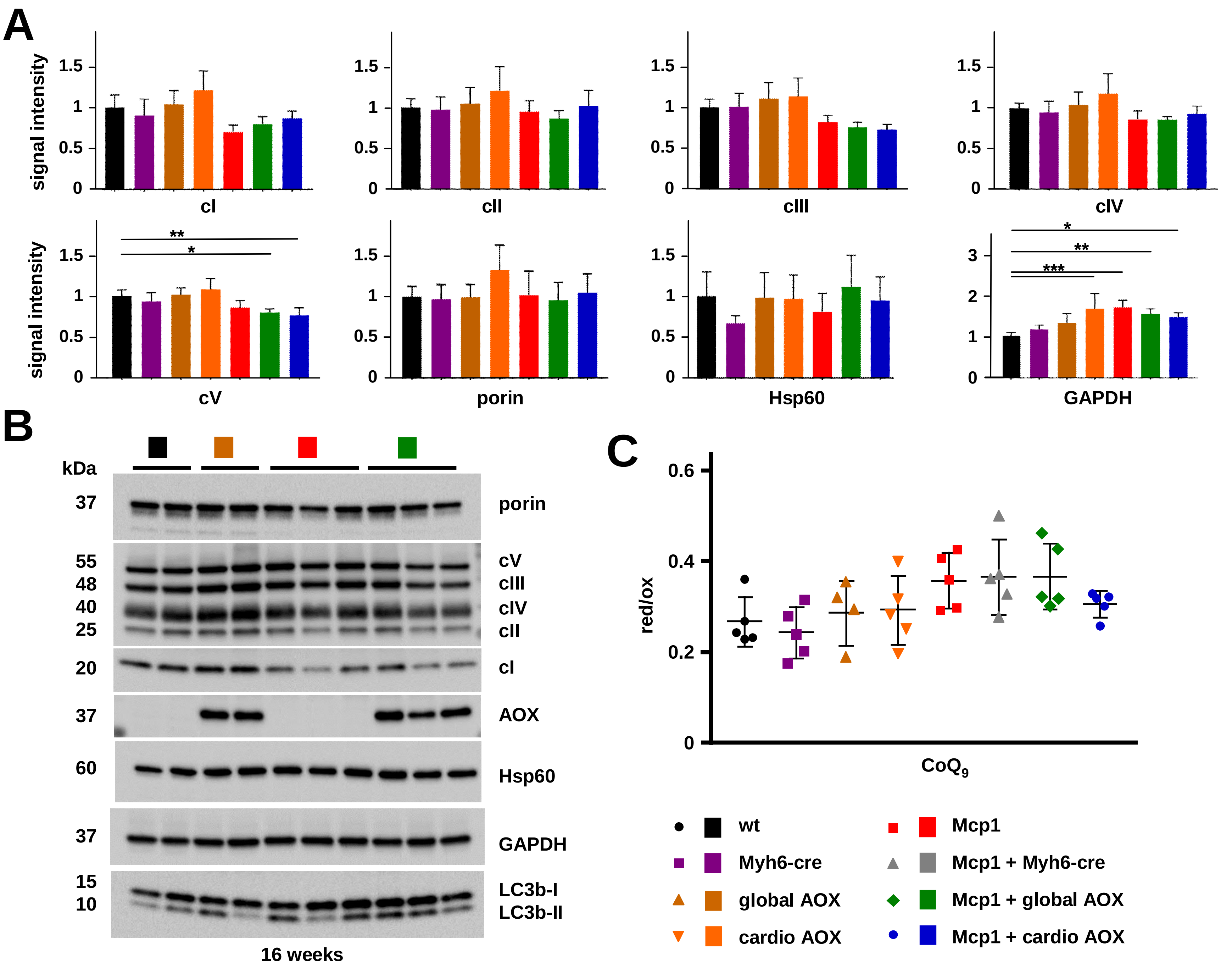

Dhandapani et al, Figure S3, page 1 of 3

D

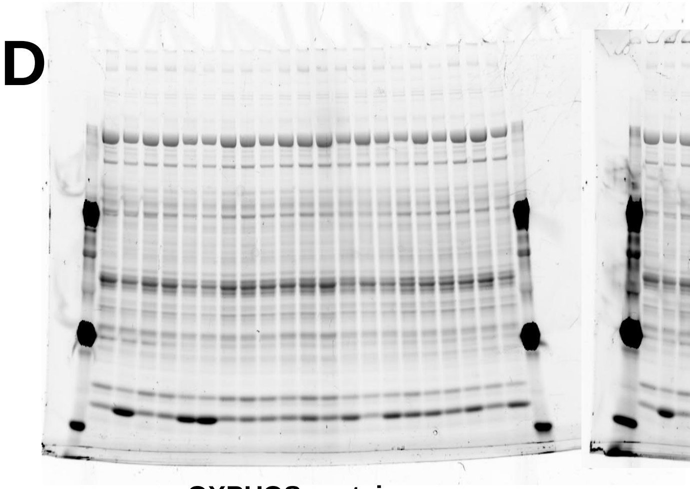

OXPHOS proteins

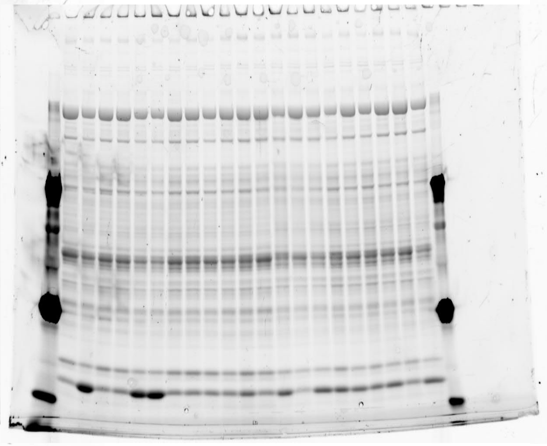

porin

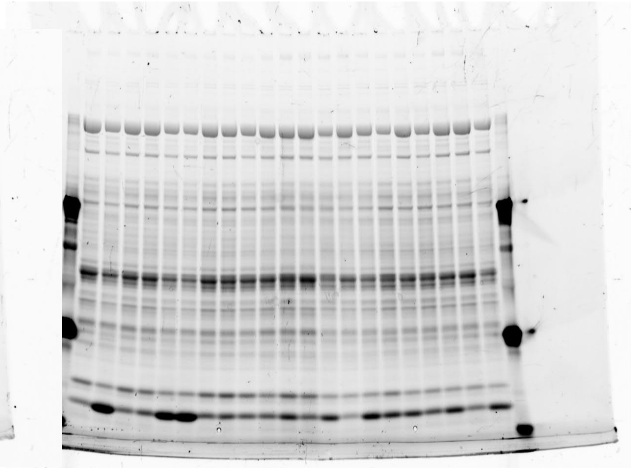

Hsp60, GAPDH

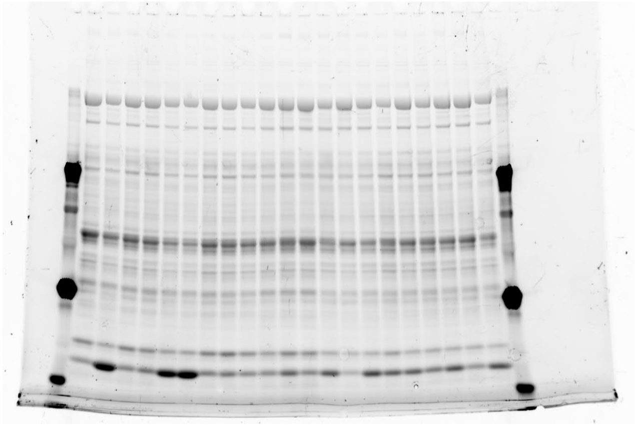

AOX, LC3b

E

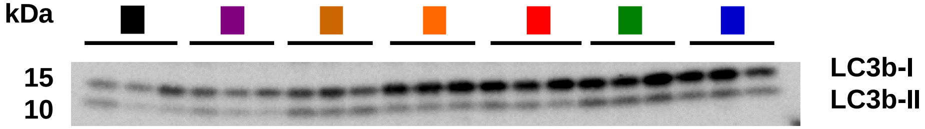

12 weeks

F

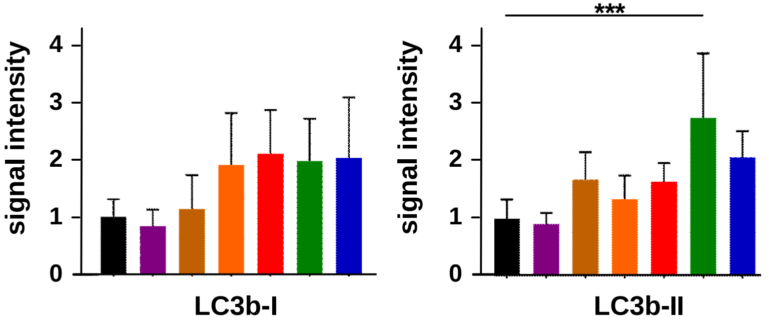

- wt
- Myh6-cre
- global AOX
- cardio AOX
- Mcp1
- Mcp1 + Myh6-cre
- Mcp1 + global AOX
- Mcp1 + cardio AOX

G

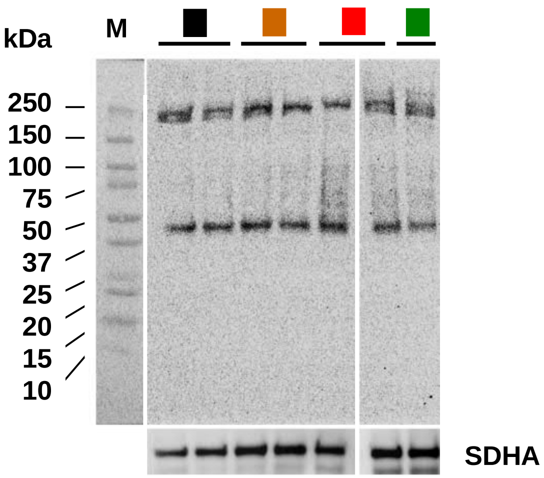

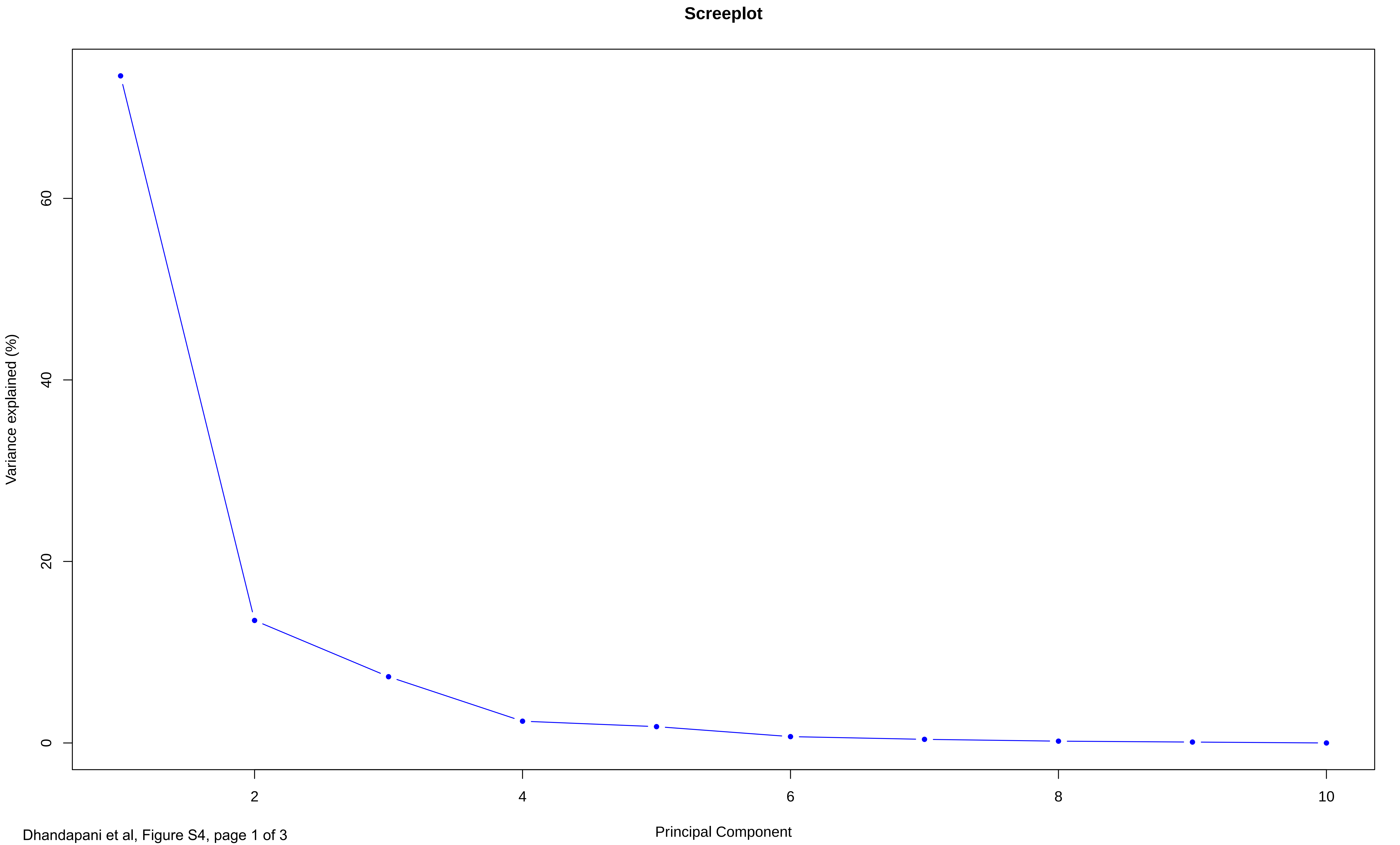
Screeplot
Variance explained (%)
0
20
40
60
2
4
6
8
10
Principal Component

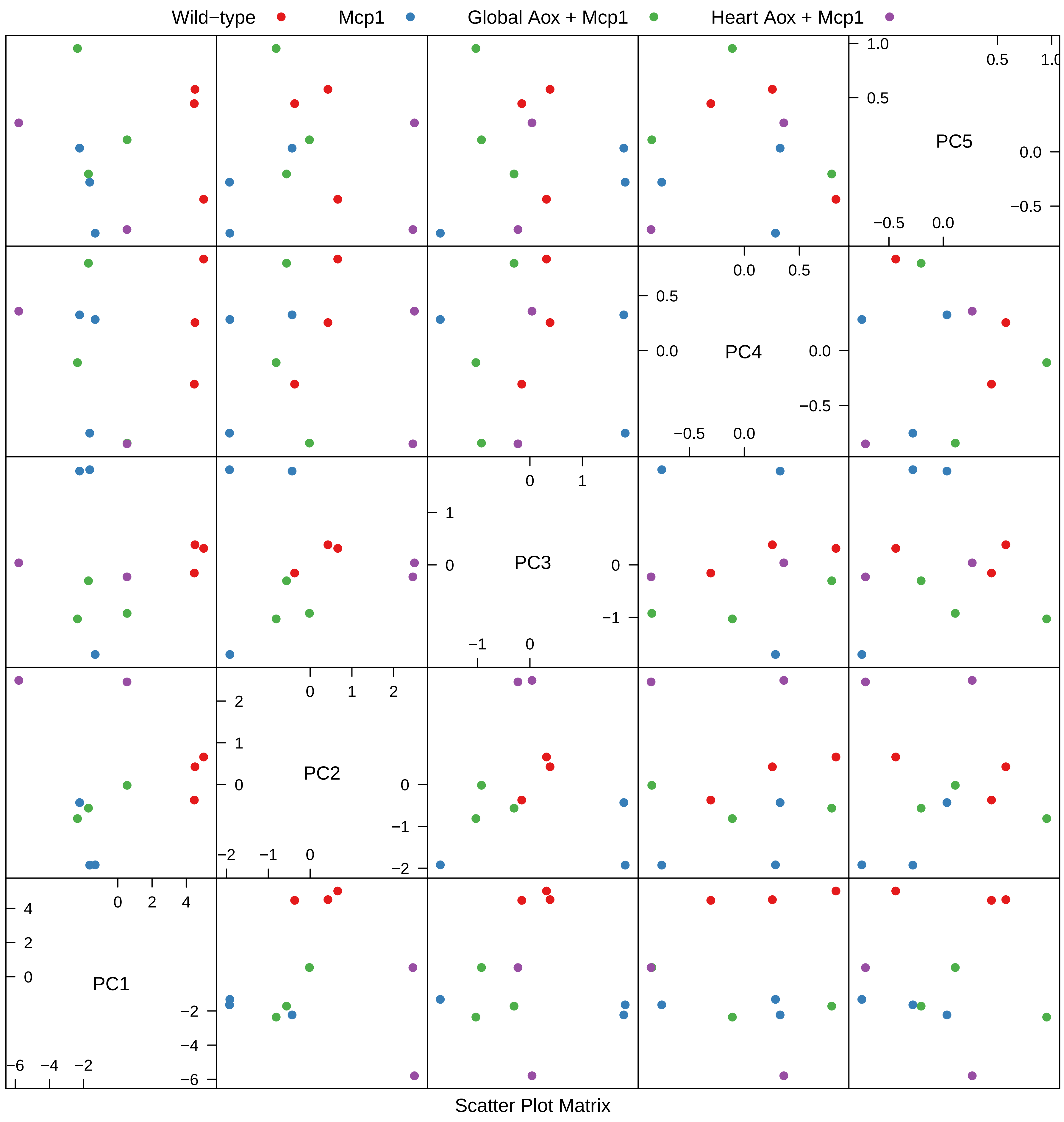

Dhandapani et al, Figure S4, page 2 of 3

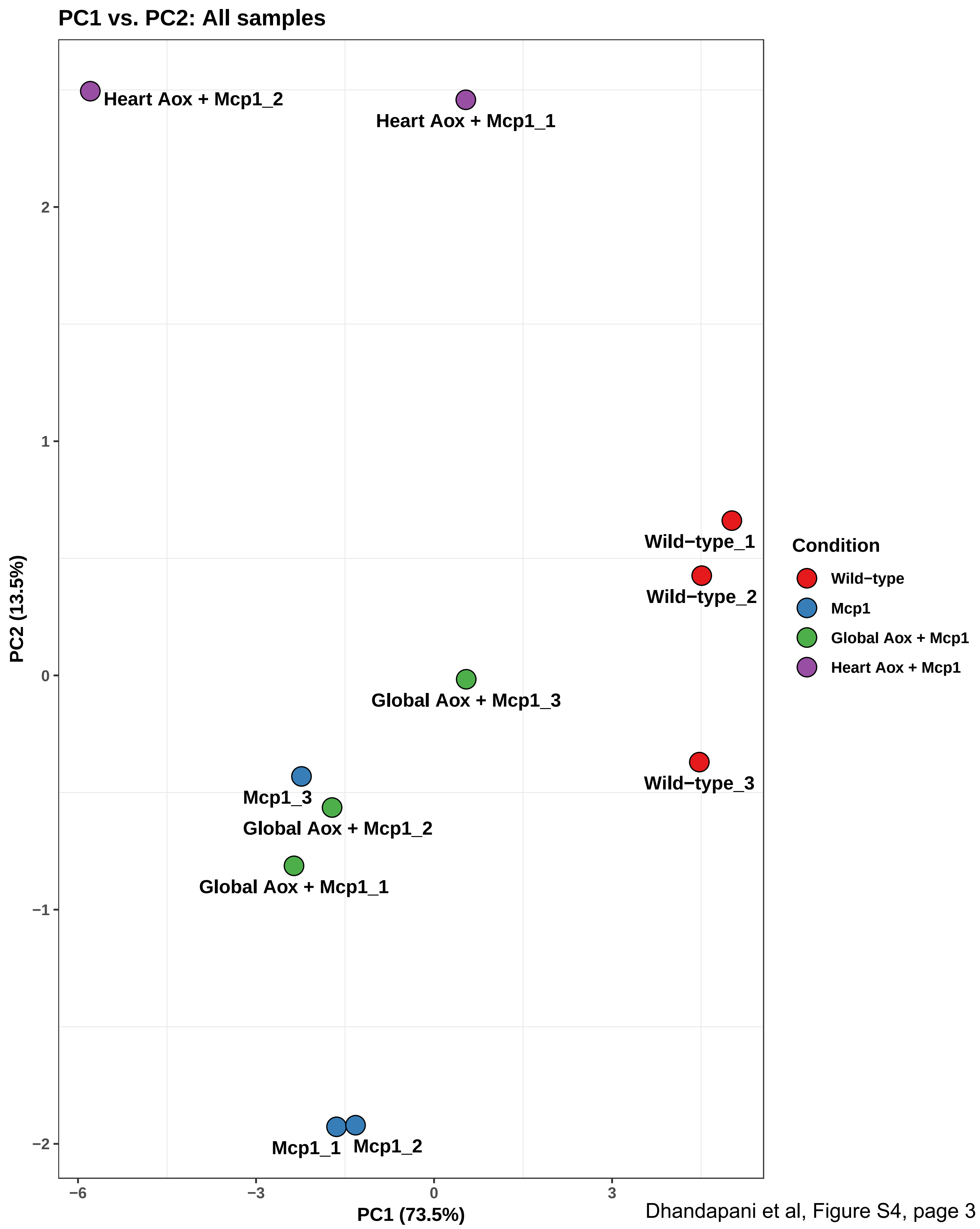
PC1 vs. PC2: All samples
Heart Aox + Mcp1_2
Heart Aox + Mcp1_1
Wild−type_1
Wild−type_2
Global Aox + Mcp1_3
Wild−type_3
Mcp1_3
Global Aox + Mcp1_2
Global Aox + Mcp1_1
Mcp1_1
Mcp1_2
PC1 (73.5%)
PC2 (13.5%)
Condition
Wild−type
Mcp1
Global Aox + Mcp1
Heart Aox + Mcp1

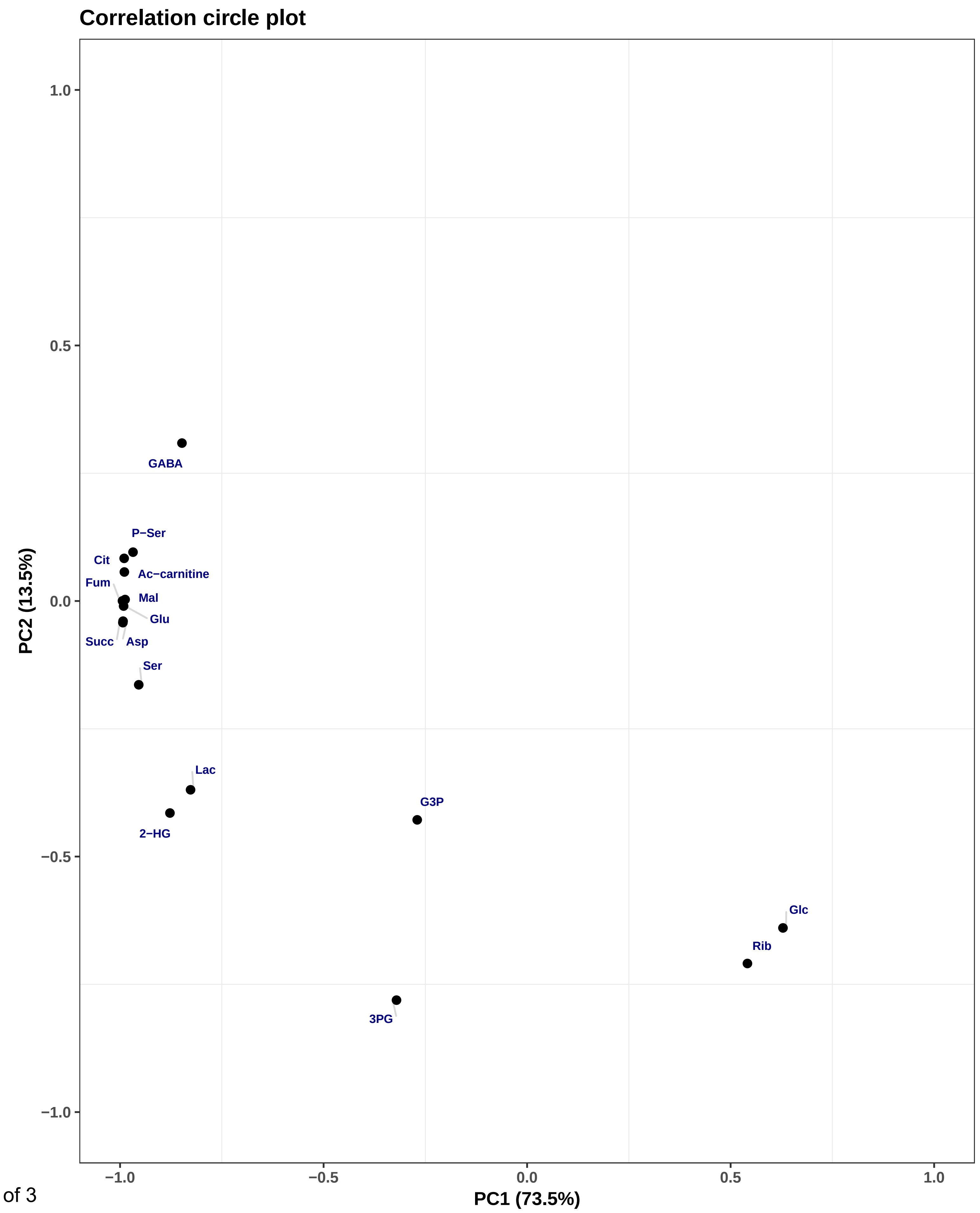
Correlation circle plot
GABA
P−Ser
Cit
Ac−carnitine
Fum
Mal
Glu
Succ
Asp
Ser
Lac
G3P
2−HG
Glc
Rib
3PG
PC1 (73.5%)
PC2 (13.5%)
